# Bioactive Indole Diketopiperazine Alkaloids from the Marine Endophytic Fungus *Aspergillus* sp. YJ191021

**DOI:** 10.3390/md19030157

**Published:** 2021-03-17

**Authors:** Jin Yang, Lizhi Gong, Miaomiao Guo, Yu Jiang, Yi Ding, Zhijie Wang, Xiujuan Xin, Faliang An

**Affiliations:** 1 Key Laboratory of Bioreactor Engineering, East China University of Science and Technology, 130 Meilong Road, Shanghai 200237, China; ecustyangjin@163.com (J.Y.); 15216603669@163.com (L.G.); jy15663497239@163.com (Y.J.); zjzsdy@163.com (Y.D.); wang56560273@163.com (Z.W.); xinxj@ecust.edu.cn (X.X.); 2 Key Laboratory of Cosmetic, Beijing Technology and Business University, China National Light Industry, 33 Fucheng Road, Beijing 100048, China; guomiaomiao7@163.com

**Keywords:** indole diketopiperazine alkaloids, endophytic fungus, *Aspergillus* sp., antimicrobial

## Abstract

Six new prenylated indole diketopiperazine alkaloids, asperthrins A–F (**1**–**6**), along with eight known analogues (**7**–**14**), were isolated from the marine-derived endophytic fungus *Aspergillus* sp. YJ191021. Their planar structures and absolute configurations were elucidated by HR-ESI-MS, 1D/2D NMR data, and time-dependent density functional theory (TDDFT)/ECD calculation. The isolated compounds were assayed for their inhibition against three agricultural pathogenic fungi, four fish pathogenic bacteria, and two agricultural pathogenic bacteria. Compound **1** exhibited moderate antifungal and antibacterial activities against *Vibrio* *anguillarum, Xanthomonas oryzae* pv. *Oryzicola*, and *Rhizoctonia* *solani* with minimal inhibitory concentration (MIC) values of 8, 12.5, and 25 μg/mL, respectively. Furthermore, **1** displayed notable anti-inflammatory activity with IC_50_ value of 1.46 ± 0.21 μM in *Propionibacterium* *acnes* induced human monocyte cell line (THP-1).

## 1. Introduction

Endophytic fungi refer to microorganisms that spend their entire or part of their life cycle in plant tissues, animals, and environments without causing any obvious infection or visible disease to the host [1]. Endophytic fungi are prolific microbial resources for the production ability of many biologically active secondary metabolites, which can help the host to resist pathogenic microorganisms [2]. Various endophytic fungi have drawn substantial attention due to their potential to produce chemically diverse and biologically active secondary metabolites with anti-cancer, anti-microbial, anti-viral, and insecticidal activities [3,4,5,6]. In our continuous searching for novel bioactive secondary metabolites from marine endophytic fungi [7,8,9], the *Aspergillus* sp. YJ191021 attracted our attention, not only for the characteristic indole diketopiperazine ultraviolet (UV) absorptions of the crude extracts, but also for their potent antimicrobial activities against agricultural pathogenic fungi.

Diketopiperazine alkaloids are valued not only for their properties and functions in fungal self-biology, but also for niche establishment to defend abiotic and biotic stress in nature. They are cyclodipeptides formed by condensation of two amino acids under the control of NRPS genes [10], especially those isolated from the genera *Aspergillus* and *Penicillium* [11]. Among them, those derived from tryptophan and proline are the most popular types in the current study, especially in structural diversity, chemical synthesis, and pharmacological activity [12,13,14]. Besides, the substitution of the isopentenyl group enriches the variability of their structures. Prenylated indole alkaloids have been reported to show a wide array of biological activities including antimicrobial, insecticidal, and cytotoxic activities [14,15]. The fascinating structural and biological properties of prenylated indole alkaloids make it possible for them to be developed into our armor and weaponry: Natural agrochemicals and drugs.

**Figure 1:**
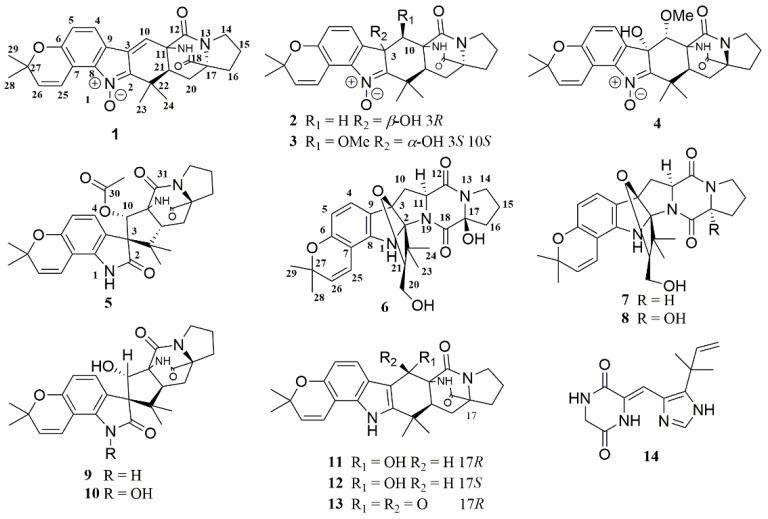
Structures of **1**–**14**.

In this study, we described the isolation and structure identification of six new prenylated indole diketopiperazine alkaloids (**1**–**6**), together with eight known analogues: Gartryprostatin A (**7**) [15], gartryprostatin B (**8**) [15], sclerotiamide (**9**) [16], notoamide H (**10**) [17], 6-epi-notoamide R (**11**) [18], notoamide R (**12**) [19], (-)-notoamide I (**13**) [20], and gartryprostatin C (**14**) [15] (Figure 1) from the marine-derived endophytic fungus *Aspergillus* sp. YJ191021. All compounds were assayed for their inhibition against three agricultural pathogenic fungi, *Rhizoctonia solani*, *Fusarium oxysporum*, *Colletotrichum gloeosporioides* penz, and two agricultural pathogenic bacteria *Xanthomonas oryzae* pv. *Oryzae*, and *X*. *oryzae* pv. *oryzicola*. Furthermore, part of the compounds was evaluated for their anti-inflammatory activity in *Propionibacterium acnes*-stimulated THP-1 human monocytic cell line. Herein, we described the isolation, structural identification, and biological evaluation of the isolated diketopiperazine alkaloids. 

## 2. Results and Discussion

Asperthrin A (**1**) was isolated as brilliant yellowish powders. Based on the [M + H]^+^ ion peak at *m*/*z* 446.2071 (calcd. for C_26_H_28_N_3_O_4_, 446.2074) in the HR-ESI-MS data and ^13^C NMR data, its molecular formula was determined as C_26_H_27_N_3_O_4_, indicating 15 degrees of unsaturation. For the NMR data (Table 1), the characteristic signals were attributed based on careful analyses of ^1^H NMR, ^13^C NMR, and HSQC spectra (Figures S1–S4) as follows: Four methyl groups at *δ*_H_ 1.22 (3H, s, Me-23), 1.55 (3H, s, Me-24), 1.42 (3H, s, Me-28), 1.41 (3H, s, Me-29), one methine proton signal at *δ*_H_ 2.31 (1H, dd, *J* = 10.2, 5.6 Hz, H-21), five olefinic protons at *δ*_H_ 7.80 (1H, d, *J* = 8.0 Hz, H-4), 6.88 (1H, d, *J* = 8.0 Hz, H-5), 7.05 (1H, s, H-10), 7.74 (1H, d, *J* = 10.2 Hz, H-25), 5.96 (1H, d, *J* = 10.2 Hz, H-26), one exchangeable proton at *δ*_H_ 8.82 (1H, s, NH-19), as well as eight aliphatic protons at *δ*_H_ 1.83–3.40 (8H, m, H_2_-14, H_2_-15, H_2_-16, H_2_-20) attributable to four methylene groups. The ^13^C NMR and DEPT data disclosed 26 carbon signals, including four *sp*^3^ non-protonated carbons at *δ*_C_ 60.3 (C-11), 66.5 (C-17), 35.6 (C-22) [one oxygen-bearing *sp*^3^ carbon at *δ*_C_ 77.1 (C-27)], and eight *sp*^2^ non-protonated carbons (*δ*_C_ 145.4, 132.7, 155.1, 111.4, 139.6, 117.6, 168.2, and 172.1). There were odd numbers of olefinic carbon signals in the ^13^C NMR spectrum, which implied that the double bond in the indole ring was connected between the carbon atom and nitrogen atom. According to the previously introduced molecular formula, there were four oxygen atoms in compound **1**. Except for two carbonyl groups and one cyclic ether group, the last oxygen atom was deducted to form the imine-oxide group located at 1-N. The presence of six double bonds and two carbonyls accounted for eight of the 15 degrees of unsaturation, indicating the existence of a heptatomic ring system for **1**.

**Figure 2:**
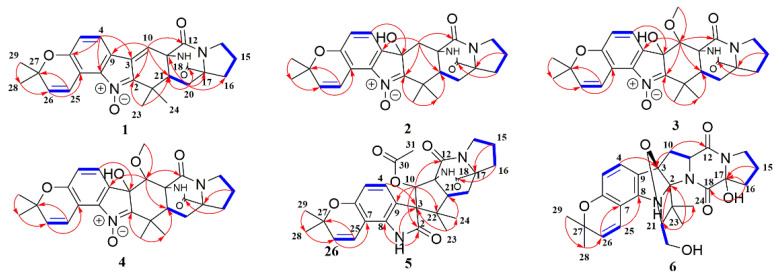
^1^H-^1^H COSY (bold blue lines) and key HMBC (red arrows) correlations for **1**–**6**.

The connection fragment of **1** was further confirmed by analysis of the HMBC spectrum (Figure S5). The HMBC correlations from H-4 (*δ*_H_ 7.80) to C-3 (*δ*_C_ 132.7)/C-6 (*δ*_C_ 155.1)/C-8 (*δ*_C_ 139.6), and H-26 (*δ*_H_ 5.96) to C-7 (*δ*_C_ 111.4)/C-27 (*δ*_C_ 77.1)/C-28 (*δ*_C_ 27.9) indicated the presence of the isopentenyl-substituted indole motif (Figure 2). The bicyclo [2.2.2]diazaoctane ring, biosynthetically derived from a diketopiperazine ring and an isoprenyl group, was indicated by key HMBC correlations from H-20a (*δ*_H_ 2.21) to C-11 (*δ*_C_ 60.3)/C-17 (*δ*_C_ 66.5)/C-18 (*δ*_C_ 172.1)/C-21 (*δ*_C_ 46.3) and from H-21 (*δ*_H_ 2.31) to C-12 (*δ*_C_ 168.2)/C-22 (*δ*_C_ 35.6)/C-23 (*δ*_C_ 17.5). Besides, the HMBC correlations from H-10 (*δ*_H_ 7.05) to C-2 (*δ*_C_ 145.4)/C-21 (*δ*_C_ 46.3) and from H-23 (*δ*_H_ 1.22) to C-2 (*δ*_C_ 145.4)/C-21 (*δ*_C_ 46.3) proved the existence of a conjugated exo-double bond-bearing cyclohexene, which was formed by the connection between prenylated indole and diazaoctane moieties. Based on the spectral analysis, the planar structure of **1** was the same as 6-*epi*-avrainvillamide, isolated from *A. taichungensis* [18]. 

**Figure 3:**
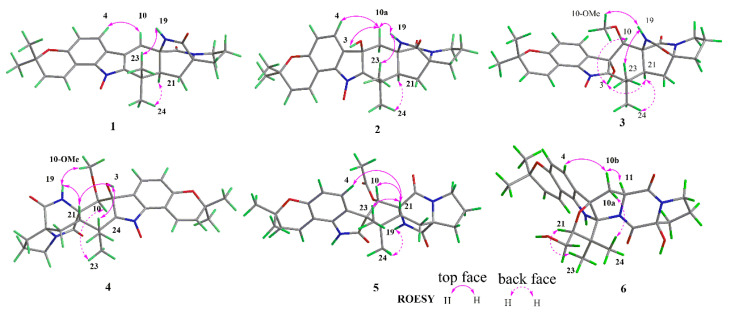
Key ROESY correlations for **1**–**6**.

**Figure 4:**
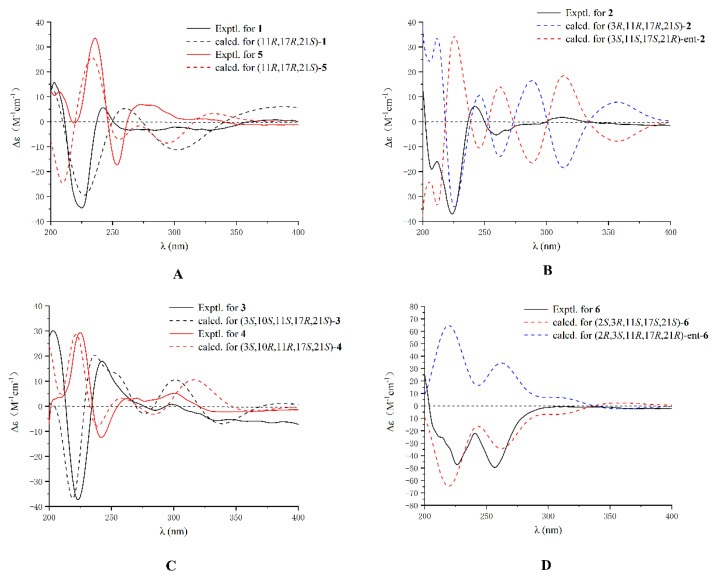
Experimental and calculated ECD spectra for **1**–**6**. (**A**) for **1** and **5**; (**B**) for **2**; (**C**) for **3** and **4**; (**D**) for **6**.

The ROESY spectrum (Figure 3 and Figure S6) exhibited correlations between 19-NH (*δ*_H_ 8.82) and H-23 (*δ*_H_ 1.22), between H-21 (*δ*_H_ 2.31) and H-24 (*δ*_H_ 1.55), supporting that H-21 and H-24 were co-facial and assigned as α-oriented whereas H-23 is β-oriented, respectively. Additionally, the absence of a cross peak between H-21 (*δ*_H_ 2.31) and 19-NH (*δ*_H_ 8.82) indicated that the relative configuration between N13-C17 and C21-22 was *anti* [21]. Williams reported that the Cotton effect at *λ* = 200–250 nm arising from an n–π* transition of the diketopiperazine moiety is diagnostic of the bicyclo[2.2.2]diazaoctane ring system [21,22]. The negative Cotton effect at 225 nm in ECD spectrum (Figure 4A and Figure S8), which was opposite to that of 6-*epi*-avrainvillamide [18], empirically indicated that the absolute configurations of C-11 and C-17 in **1** were 11*R*, and 17*R*. Combined with the analysis of the ROESY spectrum, the absolute configuration of C-21 was assigned as 21*S*. To further verify the aforementioned absolute configuration deduction of **1**, the calculated ECD spectrum was conducted. The absolute configurations of 11*R*, 17*R*, and 21*S* were determined for the well match between the calculated and the experimental ECD spectra (Figure 4A).

Asperthrin B (**2**) was obtained as white powders. The molecular formula C_26_H_29_N_3_O_5_, which was determined by the [M + Na]^+^ ion at *m*/*z* 486.2002 (calcd. for C_26_H_29_N_3_O_5_Na, 486.1999) from the HR-ESI-MS and ^13^C NMR data was 18 amu higher than the molecular mass of **1**, implying the presence of an additional hydroxy group in its structure. A careful comparison of the ^13^C NMR data of **2** with those of **1** (Table 2) showed significant upfield shifts of C-3 (*δ*_C_ 75.8) and C-10 (*δ*_C_ 36.0), indicating a saturation of the double bond between C-3 and C-10. The HMBC correlations from H-4 (*δ*_H_ 7.32) to C-3 (*δ*_C_ 75.8) (Figure 2 and Figure S14) confirmed that the hydroxyl group was attached to C-3. The ROESY correlations (Figure 3 and Figure S15) from 19-NH (*δ*_H_ 7.52) to H-23 (*δ*_H_ 1.34) and H-10a (*δ*_H_ 2.64) to 3-OH (*δ*_H_ 6.39)/19-NH (*δ*_H_ 7.52)/H-23 (*δ*_H_ 1.34) indicated these protons were co-facial and β-oriented. Accordingly, the ROESY correlations between H-21 (*δ*_H_ 2.13) and H-24 (*δ*_H_ 1.54) revealed that H-21 and H-24 were α-oriented. The absolute configurations of C-3, C-11, C-17, and C-21 in **2** were assigned as 3*R*, 11*R*, 17*R*, and 21*S* based on the negative Cotton effect at 225 nm in ECD spectra (Figure 4B and Figure S17) and calculated ECD spectra (Figure 4B).

Asperthrin C (**3**) was isolated as white powders. Based on the [M + Na]^+^ ion at *m*/*z* 516.2102 (calcd. for C_27_H_31_N_3_O_6_Na, 516.2105) in the (+)- HR-ESI-MS and ^13^C NMR data, the molecular formula was determined as C_27_H_31_N_3_O_6_, which was 30 amu more than **2**. Comparation of the ^1^H NMR spectrum (Figure S19) of **3** with that of **2** indicated that there was a methoxy group in **3**. The HMBC correlation from OMe-10 (*δ*_H_ 3.03) to C-10 (*δ*_C_ 76.9) (Figure 2 and Figure S23) suggested that the methoxy group was attached to C-10. The ROESY correlations (Figure 3 and Figure S24) between 19-NH (*δ*_H_ 7.74) and OMe-10 (*δ*_H_ 3.03)/H-23 (*δ*_H_ 1.31) indicated that these protons were co-facial and assigned as β-oriented. The ROESY correlations between 3-OH (*δ*_H_ 6.31) and H-10 (*δ*_H_ 4.72)/H-21 (*δ*_H_ 3.08), and between H-21 (*δ*_H_ 3.08) to H-24 (*δ*_H_ 1.32), indicated that they were in the α-orientation. Based on the negative Cotton effect at 225 nm in ECD spectra (Figure 4C and Figure S26) and the well match result between experimental and calculated ECD spectra (Figure 4C), the absolute configurations of C-3, C-10, C-11, C-17, and C-21 in **3** were assigned as 3*S*, 10*S*, 11*S*, 17*R*, and 21*S*.

Asperthrin D (**4**) was isolated as white powders. The molecular formula was determined as C_27_H_31_N_3_O_6_ by the (+)-HRESIMS data from the [M + Na]^+^ ion at *m*/*z* 516.2106 (calcd. for C_27_H_31_N_3_O_6_Na, 516.2105) as same as that of **3**. Detailed analyses of the 1D NMR and 2D NMR spectra (Figures S28–S32) indicated that the planar structure of **4** was the same as that of **3**. However, the chemical shifts of H-10 (*δ*_H_ 4.12) and OMe-10 (*δ*_H_ 3.31) in **4** differed from those of **3**, implying that the configuration of C-10 was opposite to that of **3**. The relative configuration was assigned by the ROESY correlations from 19-NH (*δ*_H_ 7.87) to OMe-10 (*δ*_H_ 3.31)/H-21 (*δ*_H_ 3.53), 3-OH (*δ*_H_ 6.27) to H-21 (*δ*_H_ 3.53)/H-24 (*δ*_H_ 1.30), and H-10 (*δ*_H_ 4.12) to H-23 (*δ*_H_ 1.15) (Figure 3 and Figure S33). The absolute configurations of C-3, C-10, C-11 C-17, and C-21 in **4** were assigned as 3*S*, 10*R*, 11*R*, 17*S*, 21*S* based on the positive Cotton effect at 225 nm and the calculated ECD spectra results (Figure 4C and Figure S35).

Asperthrin E (**5**) was obtained as white powders. The molecular formula was determined as C_28_H_31_N_3_O_6_ by the [M + H]^+^ ion at *m*/*z* 528.2102 (calcd. for C_28_H_31_N_3_O_6_Na, 528.2105) from HR-ESI-MS and ^13^C NMR data. The proton signal at *δ*_H_ 2.03 (3H, s) in ^1^H NMR spectrum (Figure S37) indicated the presence of an acetoxy group. The HMBC correlations from NH-1 (*δ*_H_ 10.69) to C-2 (*δ*_C_ 176.3) and H-4 (*δ*_H_ 6.89)/H-10 (*δ*_H_ 5.75)/H-23 (*δ*_H_ 0.52)/NH-1 (*δ*_H_ 10.69) to C-3 (*δ*_C_ 64.3) (Figure 2 and Figure S41) indicated the presence of an indoxyl core with a spiro-quaternary center at C-3. Detailed analyses of 1D NMR and 2D NMR spectra (Figures S37–S41) indicated that the planar structure of **5** was the same as that of 10-*O*-acetylsclerotiamide [23]. The ROESY correlations (Figure 3 and Figure S42) from 19-NH (*δ*_H_ 8.54) to H-24 (*δ*_H_ 1.12) indicated that NH-19 and H-24 were located on the α-face of the cyclopentane ring. The ROESY correlations from H-21 (*δ*_H_ 2.68) to H-10 (*δ*_H_ 5.75)/H-23 (*δ*_H_ 0.52) indicated that these protons located on the β-orientation of the cyclopentane ring. Besides, the ROESY correlations from H-21 (*δ*_H_ 2.68) to H-4 (*δ*_H_ 6.89) indicated that the cyclopentane ring was orthogonal to the plane of the indoxyl ring. Based on the positive Cotton effect at 225 nm and the calculated ECD spectra results (Figure 4A and Figure S44), the absolute configurations of C-3, C-10, C-11, C-17, and C-21 in **5** were assigned as 3*R*, 10*S*, 11*R*, 17*S*, 21*R*.

Asperthrin F (**6**) was isolated as white powders, and the molecular formula was determined as C_26_H_31_N_3_O_6_ by (+)-HRESIMS [M + Na]^+^ ion at *m*/*z* 504.2103 (calcd. for C_26_H_31_N_3_O_6_Na, 504.2105) and ^13^C NMR data, indicating 13 degrees of unsaturation. The ^1^H NMR, ^13^C NMR, and HSQC spectra (Table 1, Table 2 and Figures S46–S49) showed four methyl groups, five sp^3^-methylenes (including one oxygen-bearing methylene), two *sp*^3^-methine carbon signals (including one oxygenated carbon), five *sp*^3^ non-protonated carbons (including three oxygen-bearing carbons), four olefinic methines, and six *sp*^2^ non-protonated carbons. The ^1^H and ^13^C NMR data of **6** resembled those of gartryprostatin B [15], with the exception that C-17 oxygen-bearing sp^3^-methine signal (*δ*_C_ 93.6) and C-16 *sp*^3^-methylene signal (*δ*_H_ 2.10, 2.33; *δ*_C_ 31.6) had a clear difference. With six degrees of unsaturation accounting for the eight aromatic carbons and two carbonyls, there must be a heptatomic ring system to meet the 13 degrees of unsaturation in **6**. The HMBC correlations from H-21 (*δ*_H_ 3.81) to C-2 (*δ*_C_ 98.6), H-23 (*δ*_H_ 1.29) to C-2 (*δ*_C_ 98.6)/C-21 (*δ*_C_ 91.1)/C-24 (*δ*_C_ 18.2) (Figure 2 and Figure S50), and the deshielded shifts of C-3 (*δ*_C_ 97.1)/C-21 (*δ*_C_ 91.1) indicated that the furan ring was formed by an oxygen bridge between C-3 and C-21. Further detailed 1D and 2D NMR spectral analysis revealed that a planar structure of **6** was the same as that of gartryprostatin B. The relative configuration of C-2/C-3/C-21 in the furan ring in **6** was determined by ROESY correlations from H-21 (*δ*_H_ 3.81) to H-23 (*δ*_H_ 1.29), H-10b (*δ*_H_ 2.72) to H-4 (*δ*_H_ 6.97), and H-24 (*δ*_H_ 0.82) to H-10a (*δ*_H_ 2.59). Besides, the ROESY correlation from H-10b (*δ*_H_ 2.72) to H-11 (*δ*_H_ 4.59) determined the relative configurations of C-11. Based on the chemical shift differences of C-17 in ^13^C NMR data, the relative configurations of C-17 should be opposite to that of gartryprostatin B. The absolute configurations of C-2, C-3, C-11, C-17, and C-21 in **6** were determined as 2*S*, 3*R*, 11*S*, 17*S*, 21*S* by the comparison between calculated and experimental ECD spectra (Figure 4D and Figure S53).

Compounds **1**–**14** were assayed for their anti-agricultural pathogenic and anti-inflammatory activities. As shown in Table 3, **1** displayed both antibacterial and antifungal activities with minimal inhibitory concentration (MIC) values of 50, 12.5, and 100 μg/mL against *X*. *oryzae* pv. *oryzae*, *X*. *oryzae* pv. *oryzicola*, and *R*. *solani*, respectively. Furthermore, **1** also exhibited moderate antibacterial activity against four fish pathogens, *Edwardsiella tarda*, *Vibrio anguillarum*, *Aeromonas hydrophilia*, and *V*. *parahaemolyticus*, with MIC values of 16, 8, 32, and 16 μg/mL, respectively. Compounds **5**, **9**, and **10** showed antifungal activities with the MIC values of 25 μg/mL against *R*. *solani*. The results showed that **1**, **5**, **6**, **9**, **10**, and **12** displayed moderate anti-inflammatory activity with IC_50_ values of 1.5, 30.5, 37.2, 41.6, 46.2, and 34.3 μM, respectively, by measuring the inhibitory effects in *P. acnes*-induced THP-1 cells (Table 4).

## 3. Materials and Methods

### 3.1. General Experimental Procedures

Optical rotations were measured using a JASCO P-1020 polarimeter (JASCO Corporation, Tokyo, Japan) in MeOH at 25 °C. UV spectra were recorded on a Shimadzu UV-1800 spectrophotometer (Shimadzu Corporation, Tokyo, Japan) in MeOH. ECD spectra were obtained by Chirascan circular dichroism spectrometers (Applied Photophysics Ltd., Leatherhead, UK). Both 1D and 2D NMR spectra were recorded on a Bruker AVIII-600 NMR spectrometer, using TMS as an internal standard. High-resolution electrospray ionization (HR-ESI-MS) was carried out with an Agilent 6529B Q-TOF instrument (Agilent Technologies, Santa Clara, CA, USA). Column chromatography was performed on silica gel (200–300 mesh, Qingdao Marine Chemical Inc., Qingdao, China) and ODS (50 µm, YMC, Kyoto, Japan) on a Flash Chromatograph System (SepaBen machine, Santai Technologies, Changzhou, China). Preparative high-performance liquid chromatography (Pre-HPLC) was performed on a Shimadzu LC-20 system (Shimadzu, Tokyo, Japan) equipped with a Shim-pack RP-C18 column (20 × 250 mm i.d., 10 µm, Shimadzu, Tokyo, Japan) with a flow rate at 10 mL/min at 25 °C.

### 3.2. Fungal Material

The fungal strain *A.* sp. YJ191021 was isolated from a soil sample, which was collected from the intertidal zone of Zhoushan, Zhejiang, China, in June 2018. The fungal strain was identified according to their morphological characteristics and by sequencing the fungal ITS region in rDNA. The fungal strain is stored in State Key Laboratory of Bioreactor Engineering laboratory of Shanghai at −80 °C.

### 3.3. Fermentation, Extraction, and Isolation

The fungus was incubated on potato dextrose agar (PDA) medium at 28 °C for 3 days. Then the grown strain was inoculated to a 250 mL Erlenmeyer flask containing 50 mL of potato dextrose broth (PDB). After 2 days of fermentation, the seed cultures were added to Erlenmeyer flasks (100 × 1000 mL), each containing 100 g of dry rice and 120 mL of distilled water, which was previously sterilized at 121 °C for 30 min. All flasks were incubated at room temperature for 30 days. After incubation, whole fermented rice medium was extracted three times using ethyl acetate (EtOAc), and then solvents were concentrated under reduced pressure to give a crude extract (193.4 g). Next, the crude extract was subjected to a macroporous resin column eluting by a gradient EtOH-H_2_O (from 30%, 50%, 70% to 100% EtOH). The 50% fraction (33.6 g) was then separated on a silica gel column eluting with a stepwise gradient of CH_2_Cl_2_-MeOH (from 25:1 to 5:1) to yield five subfractions (A–E). Fraction D (4.3 g) was further purified by an ODS column (MeCN-H_2_O, 35:65) and a semi-preparative HPLC eluting with 60% MeOH/H_2_O to yield compounds **1** (22.3 mg, *t*_R_ 30.4 min), **14** (31.4 mg, *t*_R_ 10.4 min). Fraction C (3.2 g) was further purified by an ODS column (MeCN-H_2_O, 30:70) and a semi-preparative HPLC eluting with 55% MeOH/H_2_O to yield compounds **2** (13.4 mg, *t*_R_ 34.2 min), **3** (11.2 mg, *t*_R_ 30.6 min), and **4** (9.2 mg, *t*_R_ 28.1 min). Fraction B (3.6 g) was further purified by an ODS column (MeCN-H_2_O, 40:60) and a semi-preparative HPLC eluting with 60% MeOH/H_2_O to yield compounds **6** (16.7 mg, *t*_R_ 26.8 min), **7** (4.2 mg, *t*_R_ 23.4 min), and **8** (18.3 mg, *t*_R_ 19.3 min). The fraction A was separated on an ODS column (MeCN-H_2_O, 55:45) to yield four subfractions (A1–A4). The subfraction A3 was further purified by a semi-preparative HPLC eluting with 75% MeOH/H_2_O to yield compounds **5** (6.4 mg, *t*_R_ 22.6 min), **9** (12.3 mg, *t*_R_ 24.2 min), and **10** (10.1 mg, *t*_R_ 25.6 min). The subfraction A1 was further purified by a semi-preparative HPLC eluting with 65% MeOH/H_2_O to yield compounds **11** (8.4 mg, *t*_R_ 18.4 min), **12** (7.3 mg, *t*_R_ 16.2 min), and **13** (7.6 mg, *t*_R_ 19.6 min).

Asperthrin A (**1**): Brilliant yellowish powder; [α${]}_{D}^{25}$ − 75.4 (*c* 0.1, MeOH); IR *v*_max_ 3448, 1708, 1409, 1368, 1192, 1123 cm^−1^; UV (MeOH) *λ*_max_ (log *ε*) 217 (4.00), 306 (3.65) nm; ECD (2.00 mM, MeOH) *λ*_max_ (Δε) 225 (−4.33), 242 (+0.70) nm; ^1^H and ^13^C NMR data, see Table 1 and Table 2; HRESIMS at *m*/*z* 446.2071 [M + H]^+^ (calcd. for C_26_H_28_N_3_O_4_, 446.2074).

Asperthrin B (**2**): White powder; [α${]}_{D}^{25}$ − 40.2 (*c* 0.1, MeOH); IR *v*_max_ 3398, 1693, 1404, 1117, cm^−1^; UV (MeOH) *λ*_max_ (log *ε*) 203 (4.05), 266 (3.70) nm; ECD (2.00 mM, MeOH) *λ*_max_ (Δε) 224 (−4.63), 242 (+0.77) nm; ^1^H and ^13^C NMR data, see Table 1 and Table 2; HRESIMS at *m*/*z* 486.2002 [M + Na]^+^ (calcd. for C_26_H_29_N_3_O_5_Na, 486.1999).

Asperthrin C (**3**): White powder; [α${]}_{D}^{25}$ + 16.6 (*c* 0.1, MeOH); IR *v*_max_ 3417, 2983, 1689, 1538, 1496, 1121 cm^−1^; UV (MeOH) *λ*_max_ (log *ε*) 203 (4.10), 267 (3.74) nm; ECD (2.00 mM, MeOH) *λ*_max_ (Δε) 224 (−4.63), 242 (+2.23) nm; ^1^H and ^13^C NMR data, see Table 1 and Table 2; HRESIMS at *m*/*z* 516.2102 [M + Na]^+^ (calcd. for C_27_H_31_N_3_O_6_Na, 516.2105).

Asperthrin D (**4**): White powder; [α${]}_{D}^{25}$ + 32.2 (*c* 0.1, MeOH); IR *v*_max_ 3428, 2934, 1695, 1490, 1112 cm^−1^; UV (MeOH) *λ*_max_ (log *ε*) 206 (4.09), 267 (3.76) nm; ECD (2.00 mM, MeOH) *λ*_max_ (Δ*ε*) 225 (+4.21), 242 (−1.79) nm; ^1^H and ^13^C NMR data, see Table 1 and Table 2; HRESIMS at *m*/*z* 516.2106 [M + H]^+^ (calcd. for C_27_H_31_N_3_O_6_Na, 516.2105).

Asperthrin E (**5**): White powder; [α${]}_{D}^{25}$ + 53.4 (*c* 0.1, MeOH); IR *v*_max_ 3437, 2981, 1696, 1463, 1123 cm^−1^; UV (MeOH) *λ*_max_ (log *ε*) 203 (3.96), 247 (4.03) nm; ECD (2.00 mM, MeOH) *λ*_max_ (Δ*ε*) 236 (+4.17), 253 (−2.16) nm; ^1^H and ^13^C NMR data, see Table 1 and Table 2; HRESIMS at *m*/*z* 528.2102 [M + Na]^+^ (calcd. for C_28_H_31_N_3_O_6_Na, 528.2105).

Asperthrin F (**6**): White powder; [α${]}_{D}^{25}$ − 157.4 (*c* 0.1, MeOH); IR *v*_max_ 3498, 2980, 1689, 1418, 1072 cm^−1^; UV (MeOH) *λ*_max_ (log *ε*) 237 (4.16), 288 (3.64), 338 (3.50) nm; ECD (2.00 mM, MeOH) *λ*_max_ (Δ*ε*) 226 (−4.68), 241 (−2.26) nm, 257 (−4.94); ^1^H and ^13^C NMR data, see Table 1 and Table 2; HRESIMS at *m*/*z* 504.2103 [M + Na]^+^ (calcd. for C_26_H_31_N_3_O_6_Na, 504.2105).

### 3.4. Antimicrobial Assays

Minimum Inhibitory Concentration (MIC) assays were used to assess antimicrobial activities of all isolated compounds against two agricultural pathogenic bacteria (*Xanthomonas oryzae* pv. *oryzae* and *Xanthomonas oryzae* pv. *oryzicola*) and three agricultural fungi (*Colletotrichum gloeosporioides* penz, *Fusarium oxysporum* and *Rhizoctonia solani*). Furthermore, Compound **1** was tested for antibacterial activities against four fish pathogens, *Edwardsiella tarda*, *Vibrio anguillarum*, *Aeromonas hydrophilia*, and *Vibrio parahaemolyticus*. Chloromycetin was used as a positive antibacterial control and ketoconazole was used as a positive antifungal control. The experimental procedure is detailed in the (Supporting Information (SI). All the experiments were performed in three independent replicates.

### 3.5. Anti-Inflammatory Assays

The human monocyte cell line THP-1 (Cell Bank of China Science Academy, Shanghai, China) and *Propionibacterium acnes* (ATCC6919, Xiangfu biotech, Shanghai, China) were used in anti-inflammatory experiments. The *P*. *acnes* in logarithmic growth phase was used to induce inflammation in THP-1 cells. MTT method was carried out for tested compounds to determine their safe concentration to THP-1 cells. Besides, antimicrobial assays were performed to exclude false anti-inflammatory activity of these compounds raised from their inhibition to *P. acnes*. The inhibitory activity of the test compounds on the secretion of inflammatory factor 1L-1β by THP-1 cells was assayed by ELISA experiment [24,25]. The experimental procedure is detailed in the SI. All the experiments were performed in three independent replicates.

## 4. Conclusions

In summary, six new prenylated indole diketopiperazine alkaloids, asperthrins A-F (**1–6**), along with eight known analogues (**7**–**14**), were isolated from the solid rice cultures of the marine-derived fungus *Aspergillus* sp. YJ191021. Asperthrin A (**1**) showed potent anti-bacterial, anti-fungal, and anti-inflammatory activities at micromolar level. These results expand the chemical diversity of prenylated indole alkaloids and provide a basis for further development of prenylated indole alkaloids into natural agrochemicals and drug leads.

## Figures and Tables

**Table 1:** ^1^H (600 MHz) NMR Data of **1**–**6**.

Position	1 ^a^	2 ^a^	3 ^a^	4 ^a^	5 ^a^	6 ^b^
	*δ*_H_ (*J* in Hz)	*δ*_H_ (*J* in Hz)	*δ*_H_ (*J* in Hz)	*δ*_H_ (*J* in Hz)	*δ*_H_ (*J* in Hz)	*δ*_H_ (*J* in Hz)
1(NH)					10.69 (s)	
4	7.80 (d, 8.0)	7.32 (d, 8.0)	7.43 (d, 8.0)	7.36 (d, 8.1)	6.89 (d, 8.1)	6.97 (d, 8.1)
5	6.88 (d, 8.0)	6.85 (d, 8.0)	6.83 (d, 8.0)	6.88 (d, 8.1)	6.36 (d, 8.1)	6.20 (d, 8.1)
10	7.05 (s)	a 2.64 (d, 15.2)	4.72 (s)	4.12 (d, 1.2)	5.75 (s)	a 2.72 (dd, 12.9, 7.0)
		b 2.05 (d, 15.2)				b 2.59 (m)
11						4.59 (dd, 11.9, 7.0)
14	a 3.40 (m)	a 3.34 (m)	3.36 (m)	3.40 (t, 6.5)	a 3.40 (m)	3.56 (m)
	b 3.34 (m)	b 3.29 (m)			b 3.30 (m)	
15	a 2.00 (m)	a 1.97 (m)	a 1.99 (m)	a 2.02 (m)	a 1.99 (m)	a 1.99 (m)
	b 1.84 (m)	b 1.81 (m)	b 1.81 (m)	b 1.85 (m)	b 1.86 (m)	b 1.90 (m)
16	a 2.53 (m)	a 2.50 (m)	a 2.54 (m)	a 2.54 (m)	a 2.48 (m)	a 2.33 (m)
	b 1.83 (m)	b 1.83 (m)	b 1.81 (m)	b 1.85 (m)	b 1.80 (m)	b 2.10 (m)
19(NH)	8.82 (s)	7.52 (s)	7.74 (s)	7.87 (s)	8.54 (s)	
20	a 2.21 (dd, 13.4, 10.2)	a 2.13 (m)	a 2.10 (dd, 13.2, 10.3)	a 2.02 (m)	a 2.04 (m)	a 3.64 (m)
	b 1.89 (m)	b 1.85 (m)	b 1.75 (m)	b 1.85 (m)	b 1.79 (m)	b 3.56 (m)
21	2.31 (dd, 10.2, 5. 6)	2.13 (m)	3.08 (dd, 10.3, 6.5)	3.53 (dd, 10.1, 7.8)	2.68 (dd, 10.2, 6.5)	3.81 (dd, 7.6, 2.8)
23	1.22 (s)	1.34 (s)	1.31 (s)	1.15 (s)	0.52 (s)	1.29 (s)
24	1.55 (s)	1.54 (s)	1.32 (s)	1.30 (s)	1.12 (s)	0.82 (s)
25	7.74 (d, 10.2)	7.83 (d, 10.2)	7.78 (d, 10.2)	7.76 (d, 10.1)	6.57 (d, 9.8)	6.15 (d, 9.9)
26	5.96 (d, 10.2)	5.90 (d, 10.2)	5.92 (d, 10.2)	5.93 (d, 10.1)	5.75 (d, 9.8)	5.50 (d, 9.9)
28	1.42 (s)	1.39 (s)	1.41 (s)	1.42 (s)	1.38 (s)	1.37 (s)
29	1.41 (s)	1.39 (s)	1.39 (s)	1.40 (s)	1.35 (s)	1.35 (s)
31					2.03 (s)	
3-OH		6.39 (s)	6.31 (s)	6.27 (d, 1.2)		
OMe-10			3.03 (s)	3.31 (s)		

^a^ Measured at 600 MHz (^1^H) in CDCl_3_; ^b^ measured at 600 MHz (^1^H) in DMSO-*d*_6_.

**Table 2:** ^13^C (150 MHz) NMR Data of **1**–**6**.

Position	1 ^a^	2 ^a^	3 ^a^	4 ^a^	5 ^a^	6 ^b^
	*δ*c, Type	*δ*c, Type	*δ*c, Type	*δ*c, Type	*δ*c, Type	*δ*c, Type
2	145.4, C	151.2, C	153.3, C	152.4, C	176.3, C	98.6, C
3	132.7, C	75.8, C	78.5, C	78.0, C	64.3, C	97.1, C
4	121.7, CH	121.9, CH	123.1, CH	124.2, CH	125.0, CH	125.2, CH
5	116.3, CH	116.7, CH	116.1, CH	116.9, CH	108.7, CH	108.0, CH
6	155.1, C	154.3, C	154.2, C	154.4, C	152.5, C	155.1, C
7	111.4, C	112.3, C	112.0, C	111.9, C	104.8, C	103.7, C
8	139.6, C	140.0, C	140.9, C	140.1, C	138.0, C	146.8, C
9	117.6, C	131.2, C	129.3, C	129.4, C	124.6, C	118.8, C
10	121.8, CH	36.0, CH_2_	76.9, CH	76.1, CH	74.1, CH	38.9, CH_2_
11	60.3, C	61.7, C	62.1, C	62.3, C	68.9, C	63.4, CH
12	168.2, C	168.4, C	168.7, C	168.5, C	168.1, C	167.1, C
14	44.2, CH_2_	44.0, CH_2_	44.3, CH_2_	44.4, CH_2_	43.9, CH_2_	45.3, CH_2_
15	24.4, CH_2_	24.3, CH_2_	24.6, CH_2_	24.5, CH_2_	24.9, CH_2_	21.0, CH_2_
16	29.0, CH_2_	28.9, CH_2_	29.0, CH_2_	29.1, CH_2_	28.1, CH_2_	31.6, CH_2_
17	66.5, C	66.8, C	66.9, C	66.8, C	69.3, C	93.6, C
18	172.1, C	172.4, C	172.4, C	172.2, C	172.5, C	165.4, C
20	30.5, CH_2_	31.8, CH_2_	29.2, CH_2_	30.5, CH_2_	27.9, CH_2_	61.8, CH_2_
21	46.3, CH	48.7, CH	42.0, CH	50.0, CH	51.1, CH	91.1, CH
22	35.6, C	38.6, C	36.8, C	36.6, C	47.2, C	46.7, C
23	17.5, CH_3_	21.2, CH_3_	14.3, CH_3_	13.5, CH_3_	25.6, CH_3_	21.2, CH_3_
24	23.4, CH_3_	27.0, CH_3_	22.9, CH_3_	22.8, CH_3_	21.3, CH_3_	18.2, CH_3_
25	115.8, CH	116.3, CH	116.0, CH	115.9, CH	117.2, CH	116.6, CH
26	133.7, CH	133.2, CH	133.3, CH	133.5, CH	130.8, CH	129.1, CH
27	77.1, C	76.5, C	76.6, C	76.7, C	76.3, C	76.0, C
28	27.9, CH_3_	27.8, CH_3_	27.9, CH_3_	27.9, CH_3_	28.8, CH_3_	28.2, CH_3_
29	27.9, CH_3_	27.8, CH_3_	27.8, CH_3_	27.9, CH_3_	27.9, CH_3_	27.6, CH_3_
30					170.0, C	
31					21.0, CH_3_	
10-OMe			61.8, CH_3_	60.0, CH_3_		

^a^ Measured at 150 MHz (^13^C) in CDCl_3_; ^b^ measured at 150 MHz (^13^C) in DMSO-*d_6_.*

**Table 3:** Antimicrobial activities of compounds **1**–**14** (MIC, μg/mL).

No.	Bacteria						Fungi		
	Xe	Xa	Et	Va	Ah	Vp	Rs	Fo	Cg
**1**	50	12.5	16	8	32	16	100	>100	>100
**5**	>100	>100	—	—	—	—	25	>100	>100
**9**	>100	>100	—	—	—	—	25	>100	>100
**10**	>100	>100	—	—	—	—	25	>100	>100
Chloromycetin	12.5	12.5	2	0.5	2	2	—	—	—
Ketoconazole	—	—	—	—	—	—	0.78	100	12.5

Xe: *Xanthomonas oryzae* pv. *oryzae*; Xa: *Xanthomonas oryzae* pv. *oryzicola*; Et: *Edwardsiella tarda*; Va: *Vibrio anguillarum*; Ah: *Aeromonas hydrophilia*; Vp: *Vibrio parahaemolyticus*; Rs: *Rhizoctonia solani*; Fo: *Fusarium oxysporum*; Cg: *Colletotrichum gloeosporioides*.

**Table 4:** Anti-inflammatory activities of tested compounds.

No.	THP-1 Cells		*P. acnes*
	IC_50_ (μM)	SC (μM)	MIC (μM)
**1**	1.46 ± 0.21	0–5	>5
**5**	30.5 ± 0.2	0–40	>40
**6**	37.2 ± 3.1	0–50	>50
**9**	41.6 ± 1.3	0–50	>50
**10**	46.2 ± 2.2	0–50	>50
**12**	34.3 ± 1.6	0–50	>50
Tretinoin	3.38 ± 0.28	0–50	>50

SC: Safe concentration, indicating the concentration range of THP-1 cells viability over 80% treated by tested compounds.

## Supplementary Materials

The following are available online at https://www.mdpi.com/1660-3397/19/3/157/s1, Figures S1–S54: ^1^H, ^13^C, HSQC, HMBC, ROESY, UV, IR, ECD, and HRESIMS spectra of the new compounds **1**–**6**, Tables S1–S6: Computational data of **1** and **4**.
