# Supplementary material for: Bioactive Indole Diketopiperazine Alkaloids from the Marine Endophytic Fungus Aspergillus sp. YJ191021
Source: Mar Drugs. 2021 Mar 17;19(3):157. doi: 10.3390/md19030157 (PMC8002477; doi:10.3390/md19030157)
Supplement: Supplementary file 1 [file marinedrugs-19-00157-s001.pdf]

Supporting Information

Bioactive Indole Diketopiperazine Alkaloids from the Marine Endophytic Fungus *Aspergillus* sp. YJ191021

Jin Yang[a], Lizhi Gong[a], Miaomiao Guo[b], Yu Jiang[a], Yi Ding[a], Zhijie Wang[a], Xiujuan Xin[a], Faliang An[a,]*

[a]State Key Laboratory of Bioreactor Engineering, East China University of Science and Technology, 130 Meilong Road, Shanghai 200237, People's Republic of China

[b]Key Laboratory of Cosmetic, Beijing Technology and Business University, China National Light Industry, 33 Fucheng Road, Beijing 100048, China

Contents

Figure S1 ^{1}H NMR spectrum (600 MHz) of compound **1** in DMSO-d_6........................4
Figure S2 ^{13}C NMR spectrum (150 MHz) of compound **1** in DMSO -d_6……………4
Figure S3 ^{1}H-^{1}H COSY spectrum of compound **1** in DMSO -d_6..................................5
Figure S4 HSQC spectrum of compound **1** in DMSO -d_6…………………..………5
Figure S5 HMBC spectrum of compound **1** in DMSO-d_6..6
Figure S6 ROESY spectrum of compound **1** in DMSO-d_6……………………………6
Figure S7 UV spectrum of compound **1** in MeOH 7
Figure S8 ECD spectrum of compound **1** in MeOH...7
Figure S9 HRESIMS spectrum of compound **1**...7
Figure S10 ^{1}H NMR spectrum (600 MHz) of compound **2** in DMSO-d_6.....................8
Figure S11 ^{13}C NMR spectrum (150 MHz) of compound **2** in DMSO -d_6……………8
Figure S12 ^{1}H-^{1}H COSY spectrum of compound **2** in DMSO -d_6...............................9
Figure S13 HSQC spectrum of compound **2** in DMSO -d_6……………………………9
Figure S14 HMBC spectrum of compound **2** in DMSO -d_6.......................................10
Figure S15 ROESY spectrum of compound **2** in DMSO-d_6…………………………10
Figure S16 UV spectrum of compound **2** in MeOH 11
Figure S17 ECD spectrum of compound **2** in MeOH..11
Figure S18 HRESIMS spectrum of compound **2**..11
Figure S19 ^{1}H NMR spectrum (600 MHz) of compound **3** in DMSO-d_6..................12
Figure S20 ^{13}C NMR spectrum (150 MHz) of compound **3** in DMSO -d_6…………12
Figure S21 ^{1}H-^{1}H COSY spectrum of compound **3** in DMSO -d_6.............................13
Figure S22 HSQC spectrum of compound **3** in DMSO -d_6…………………………13
Figure S23 HMBC spectrum of compound **3** in DMSO -d_6.......................................14
Figure S24 ROESY spectrum of compound **3** in DMSO-d_6…………………………14
Figure S25 UV spectrum of compound **3** in MeOH 15
Figure S26 ECD spectrum of compound **3** in MeOH..15
Figure S27 HRESIMS spectrum of compound **3**..15
Figure S28 ^{1}H NMR spectrum (600 MHz) of compound **4** in DMSO-d_6..................16
Figure S29 ^{13}C NMR spectrum (150 MHz) of compound **4** in DMSO -d_6…………16
Figure S30 ^{1}H-^{1}H COSY spectrum of compound **4** in DMSO -d_6.............................17
Figure S31 HSQC spectrum of compound **4** in DMSO -d_6…………………………17
Figure S32 HMBC spectrum of compound **4** in DMSO -d_6.......................................18
Figure S33 ROESY spectrum of compound **4** in DMSO-d_6…………………………18
Figure S34 UV spectrum of compound **4** in MeOH 19
Figure S35 ECD spectrum of compound **4** in MeOH..19
Figure S36 HRESIMS spectrum of compound **4**..19
Figure S37 ^{1}H NMR spectrum (600 MHz) of compound **5** in DMSO-d_6...................20
Figure S38 ^{13}C NMR spectrum (150 MHz) of compound **5** in DMSO -d_6…………20
Figure S39 ^{1}H-^{1}H COSY spectrum of compound **5** in DMSO -d_6.............................21
Figure S40 HSQC spectrum of compound **5** in DMSO -d_6…………………………21

Figure S41 HMBC spectrum of compound **5** in DMSO -d_6 22
Figure S42 ROESY spectrum of compound **5** in DMSO-d_6............................22
Figure S43 UV spectrum of compound **5** in MeOH 23
Figure S44 ECD spectrum of compound **5** in MeOH 23
Figure S45 HRESIMS spectrum of compound **5** 23
Figure S46 ^{1}H NMR spectrum (600 MHz) of compound **6** in $CDCl_3$ 24
Figure S47 ^{13}C NMR spectrum (150 MHz) of compound **6** in $CDCl_3$.....................24
Figure S48 ^{1}H-^{1}H COSY spectrum of compound **6** in $CDCl_3$ 25
Figure S49 HSQC spectrum of compound **6** in $CDCl_3$..25
Figure S50 HMBC spectrum of compound **6** in $CDCl_3$..26
Figure S51 ROESY spectrum of compound **6** in $CDCl_3$ 26
Figure S52 UV spectrum of compound **6** in MeOH 27
Figure S53 ECD spectrum of compound **6** in MeOH 27
Figure S54 HRESIMS spectrum of compound **6**...27
Figure S55 IR spectrum of compound **1** 27
Figure S56 IR spectrum of compound **2** 27
Figure S57 IR spectrum of compound **3** 27
Figure S58 IR spectrum of compound **4** 27
Figure S59 IR spectrum of compound **5** 27
Figure S60 IR spectrum of compound **6** 27
ECD Calculation 30
Antimicrobial Assay...30
Anti Inflammatory Assay..30
18S rDNA Sequence..31

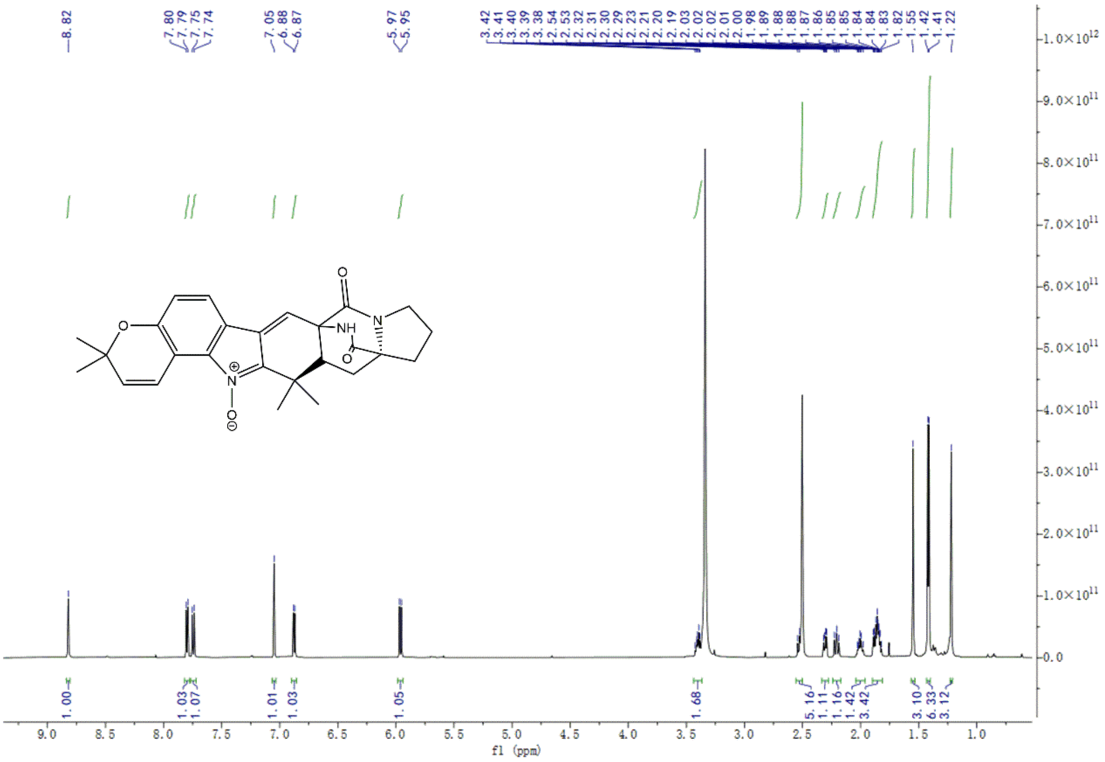

Figure S1 ^{1}H NMR spectrum (600 MHz) of compound **1** in DMSO-d_6

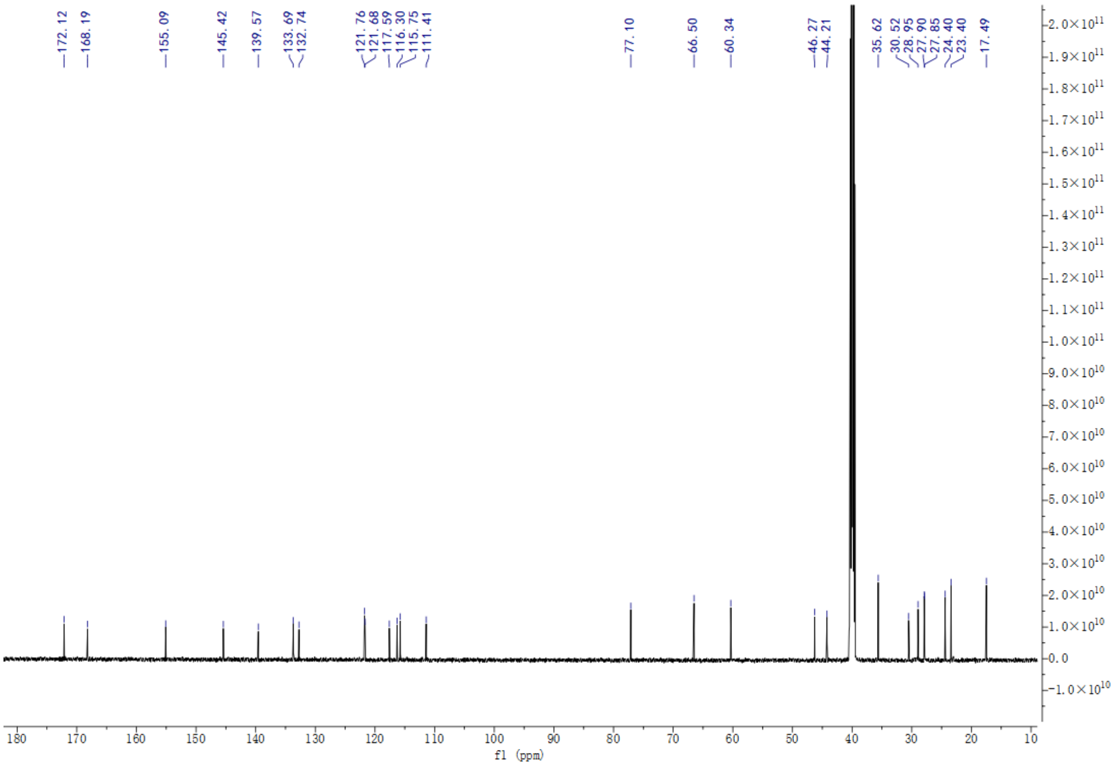

Figure S2 ^{13}C NMR spectrum (150 MHz) of compound **1** in DMSO -d_6

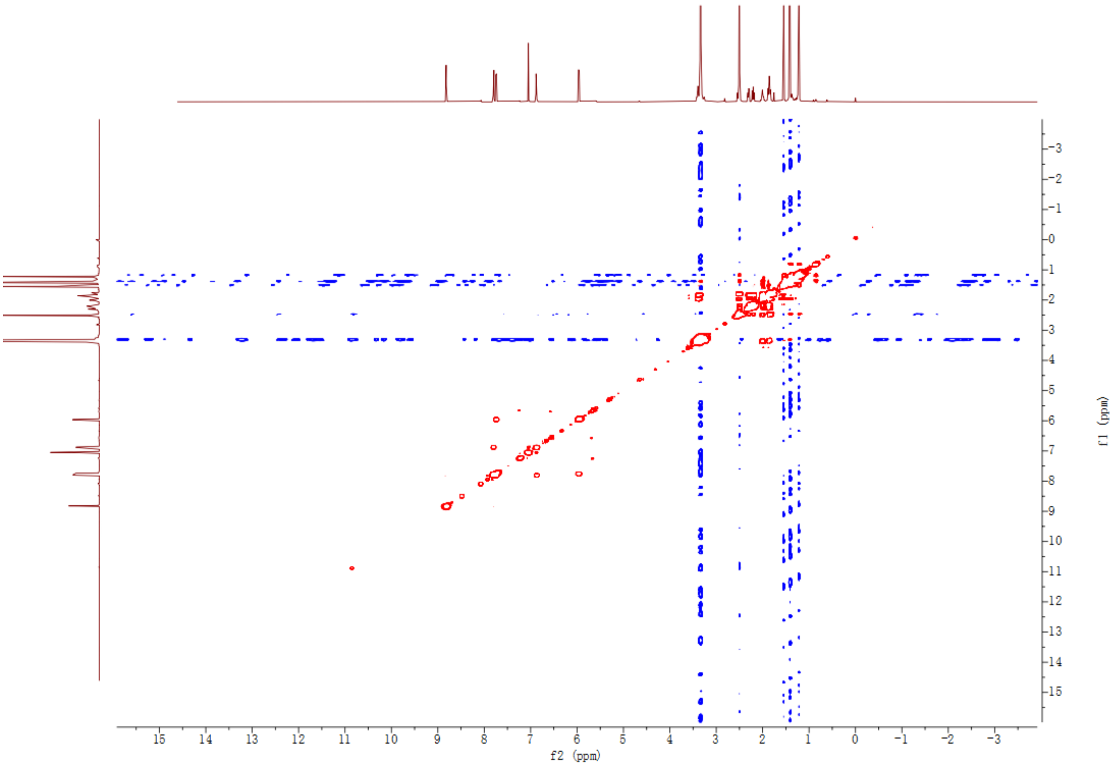

Figure S3 ^{1}H-^{1}H COSY spectrum of compound **1** in DMSO -*d*6

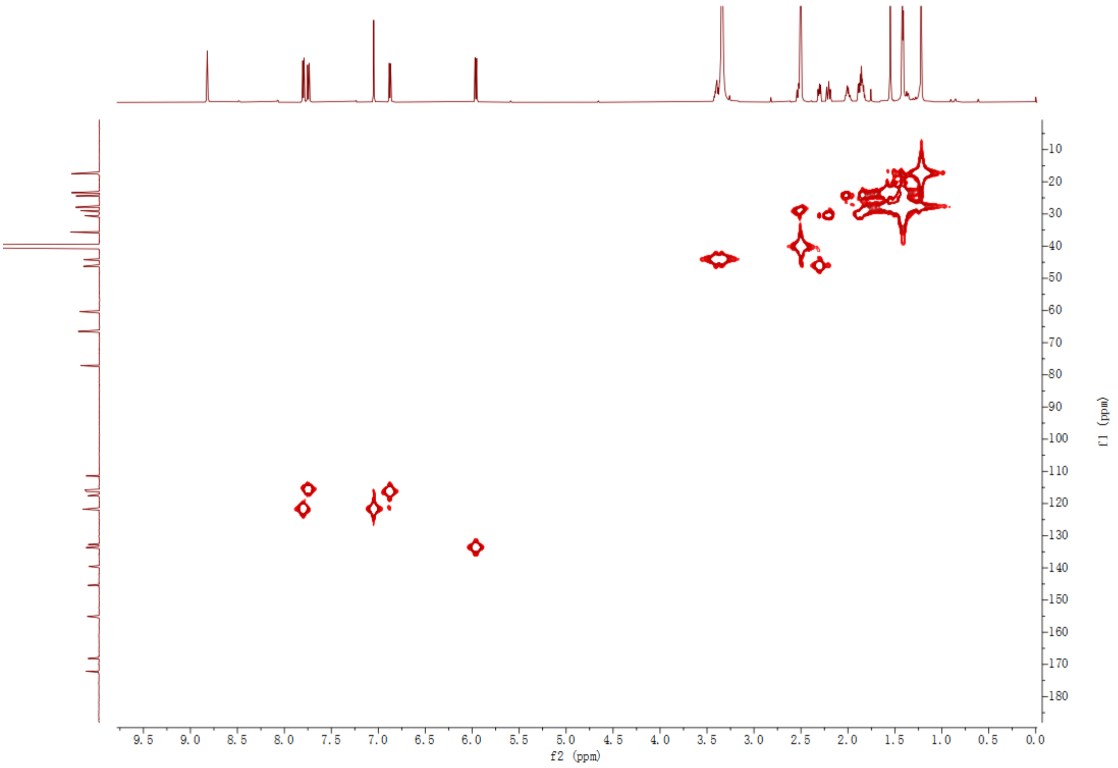

Figure S4 HSQC spectrum of compound **1** in DMSO -*d*6

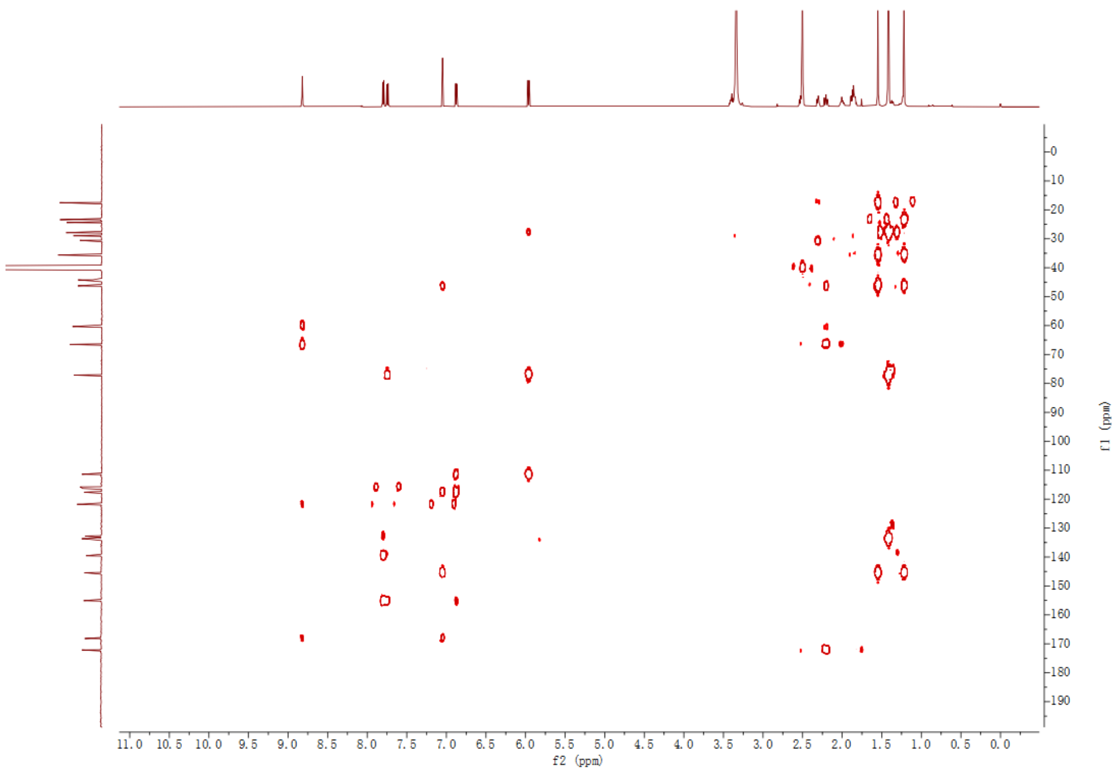

Figure S5 HMBC spectrum of compound **1** in DMSO-d_6

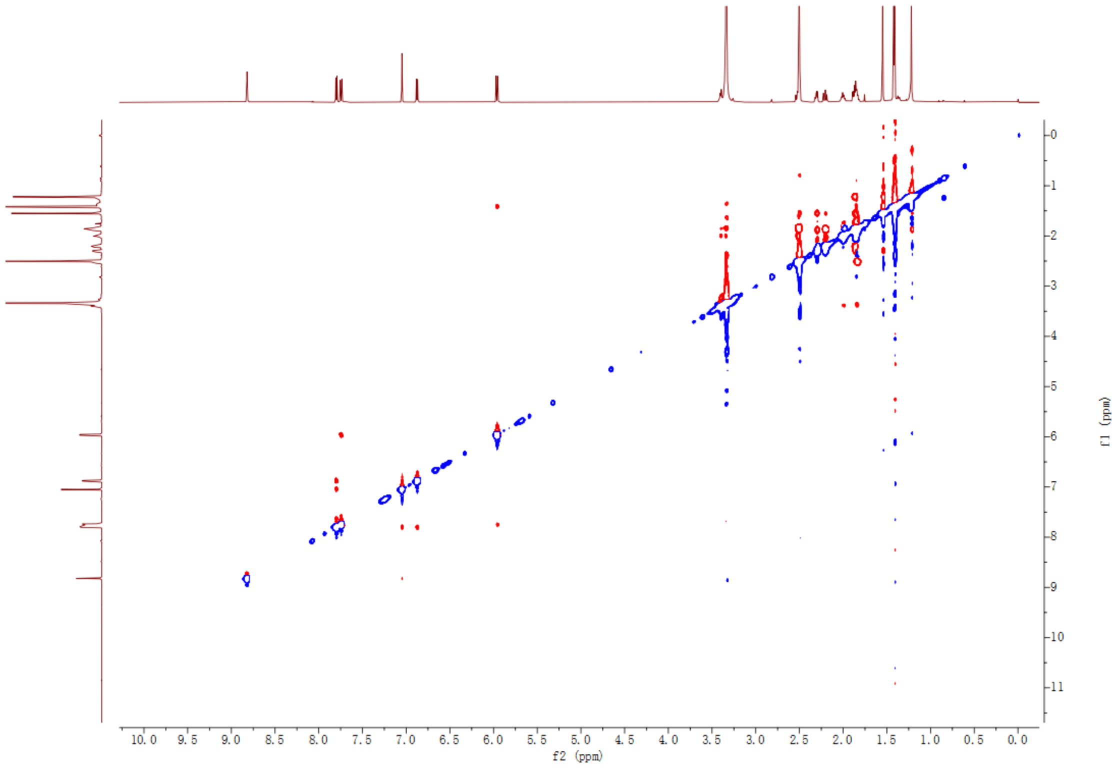

Figure S6 ROESY spectrum of compound **1** in DMSO-d_6

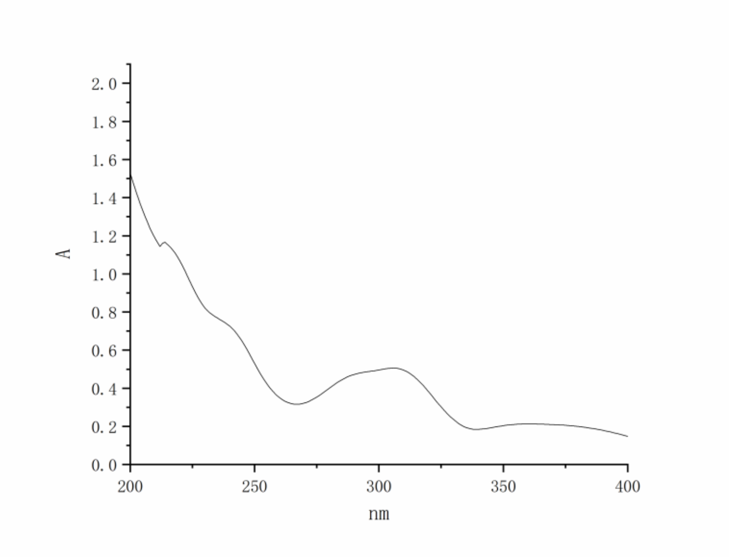

Figure S7 UV spectrum of compound **1** in MeOH

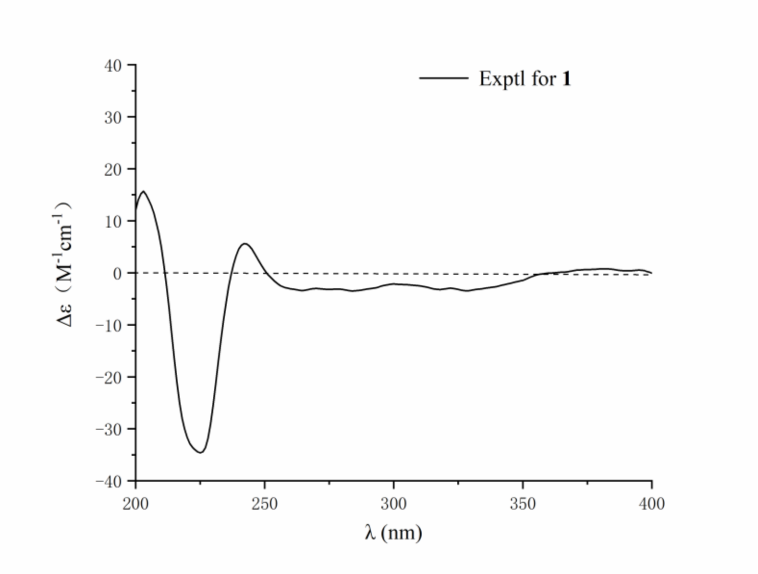

Figure S8 ECD spectrum of compound **1** in MeOH

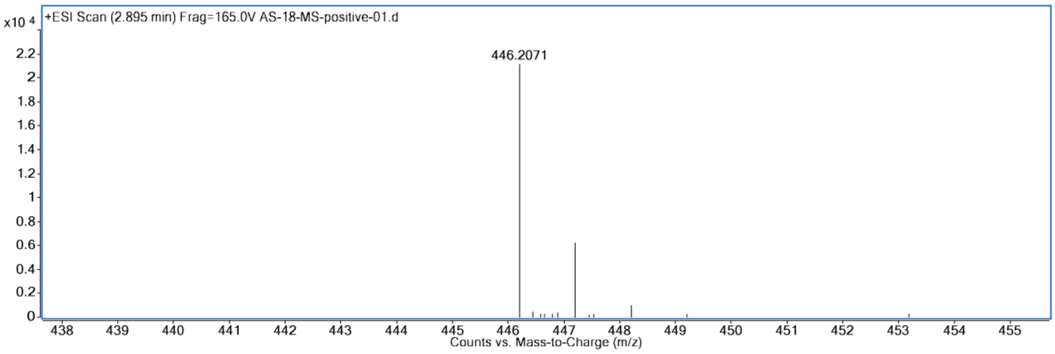

Figure S9 HRESIMS spectrum of compound **1**

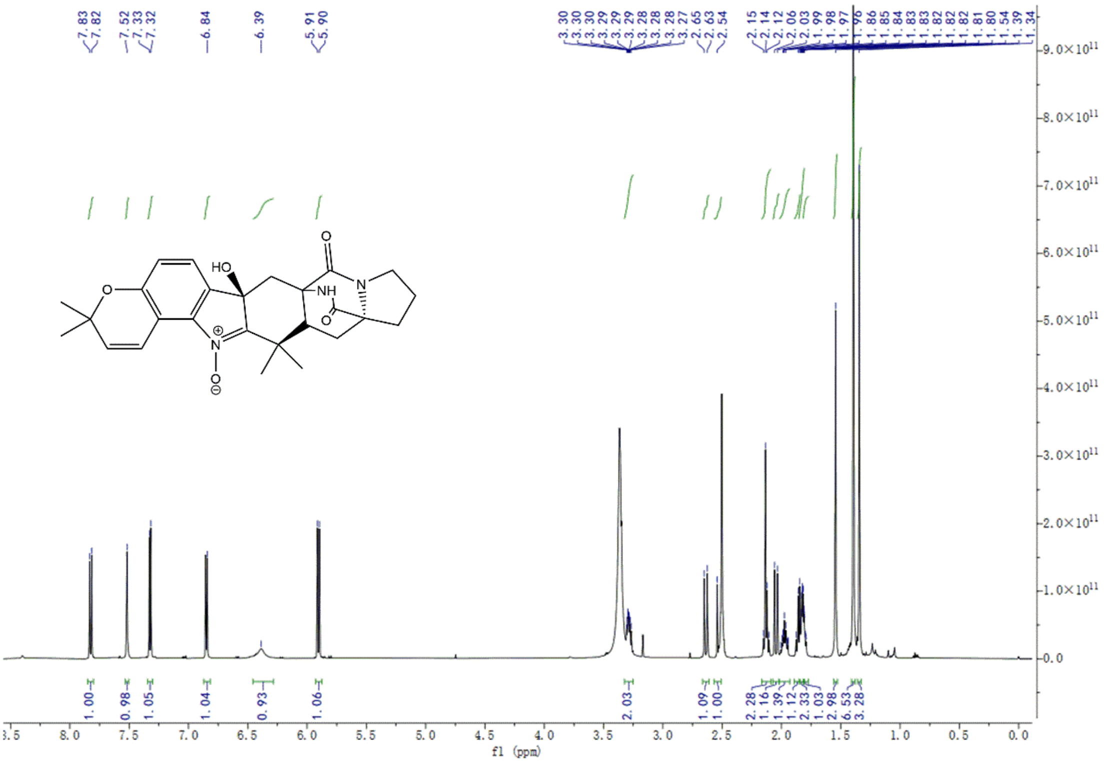

Figure S10 ^{1}H NMR spectrum (600 MHz) of compound **2** in DMSO-d_6

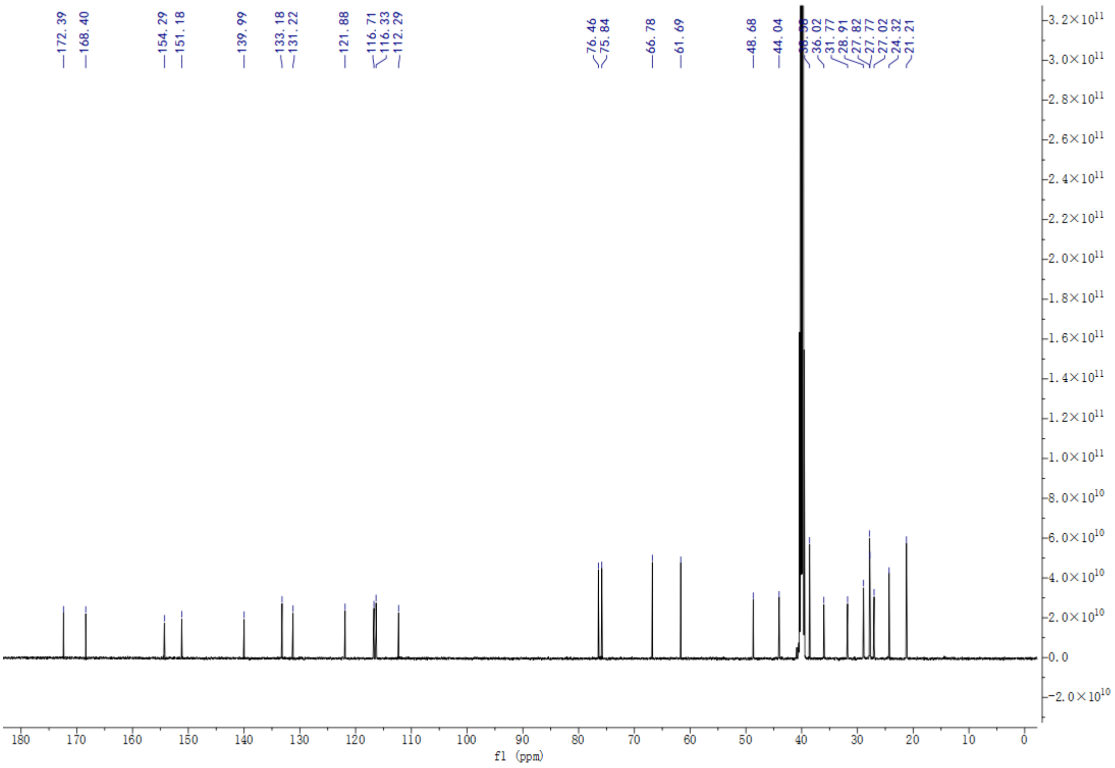

Figure S11 ^{13}C NMR spectrum (150 MHz) of compound **2** in DMSO -d_6

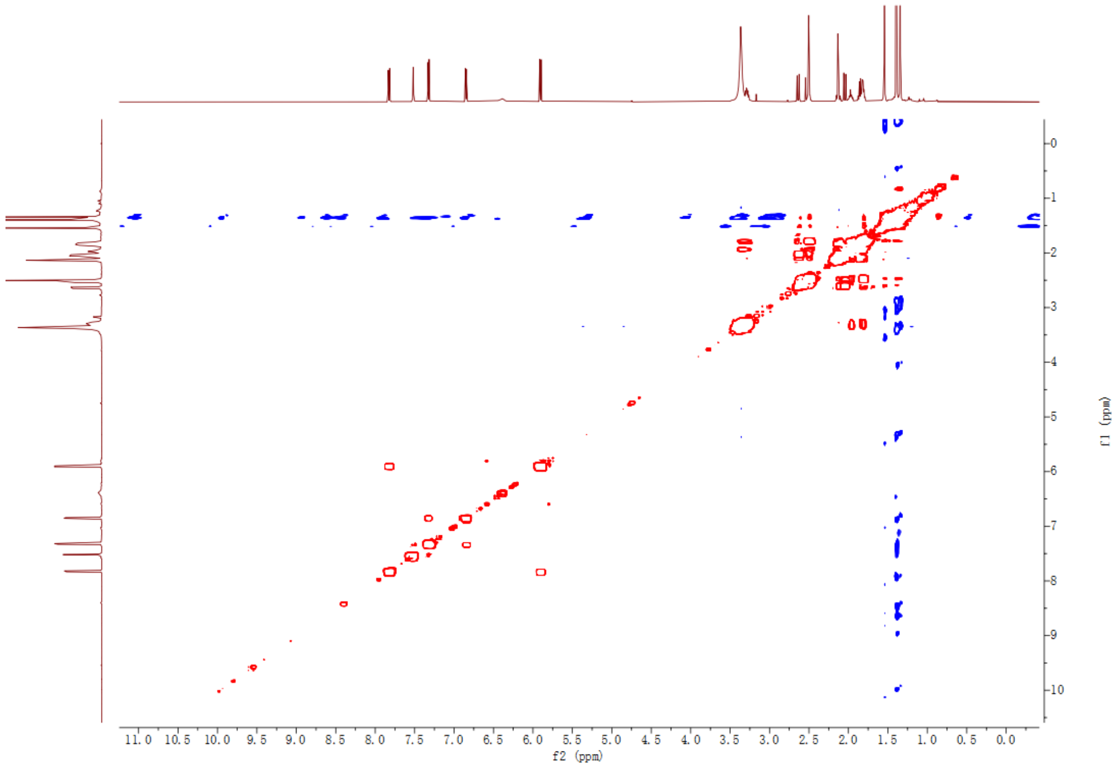

Figure S12 ^{1}H-^{1}H COSY spectrum of compound **2** in DMSO -*d*6

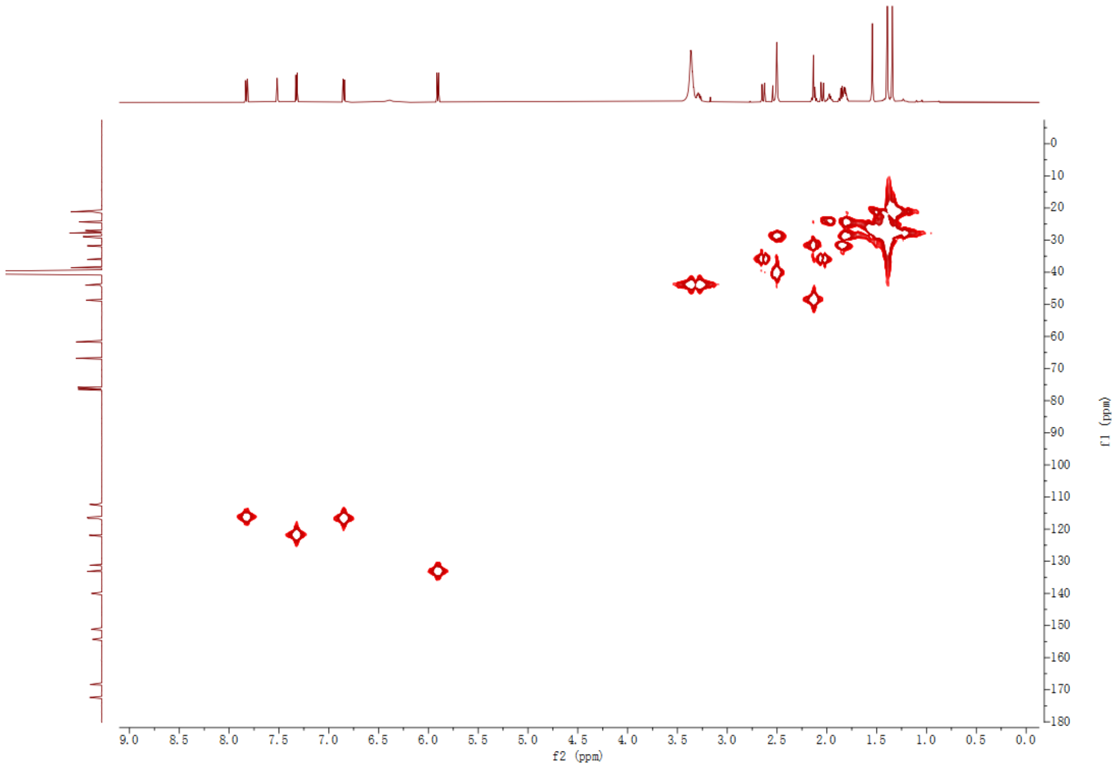

Figure S13 HSQC spectrum of compound **2** in DMSO -*d*6

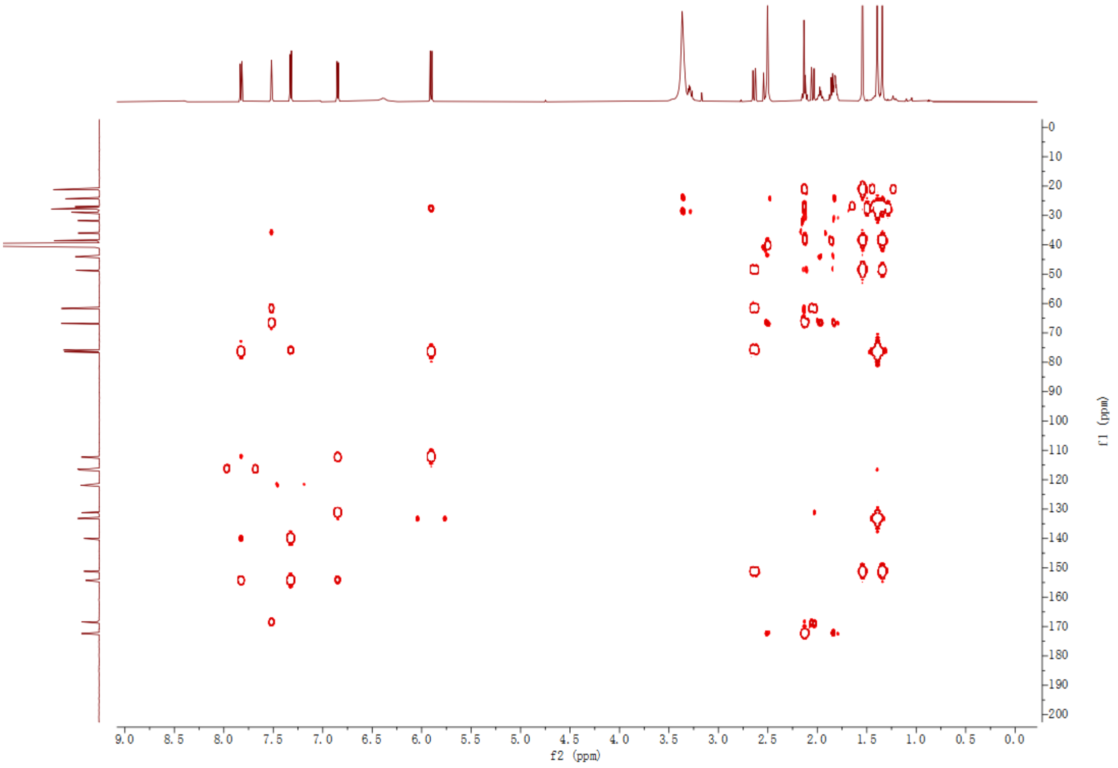

Figure S14 HMBC spectrum of compound **2** in DMSO -d_6

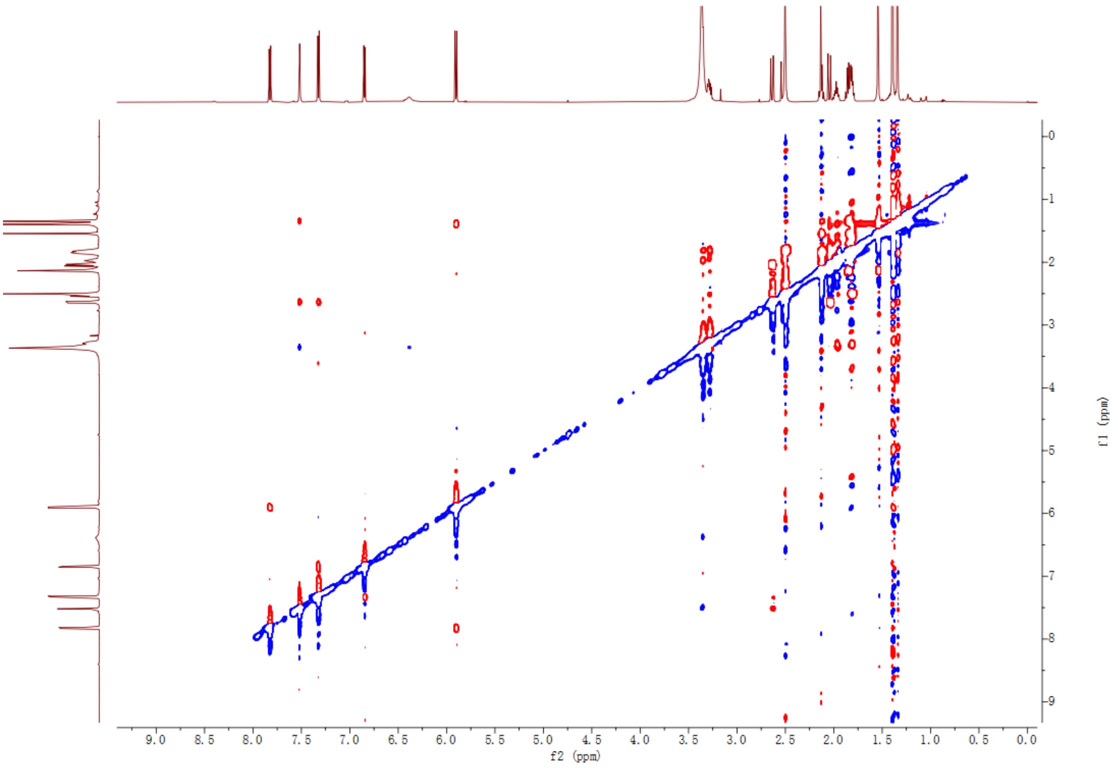

Figure S15 ROESY spectrum of compound **2** in DMSO -d_6

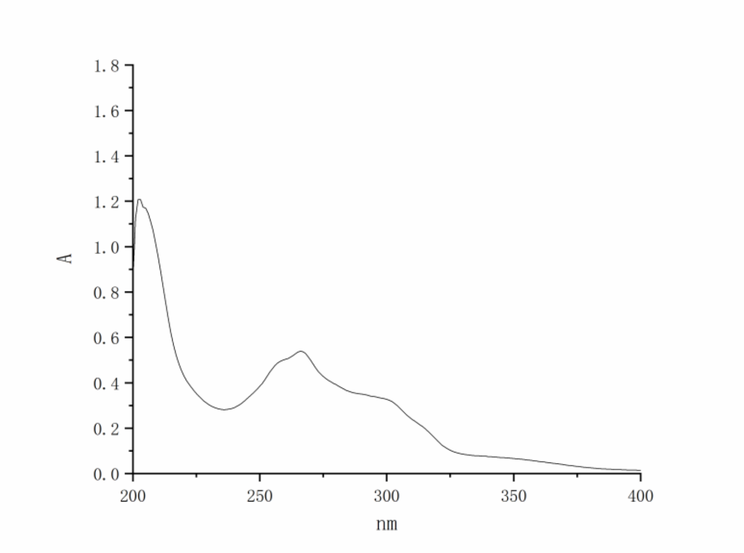

Figure S16 UV spectrum of compound **2** in MeOH

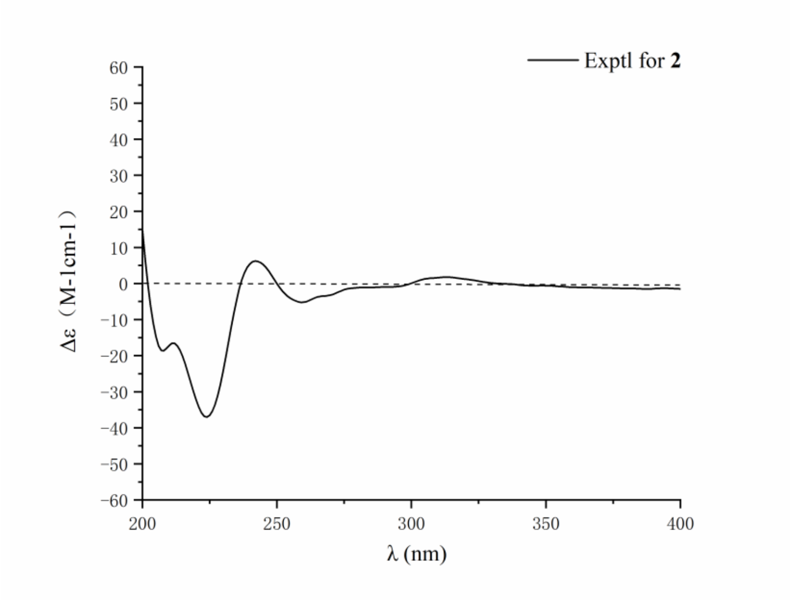

Figure S17 ECD spectrum of compound **2** in MeOH

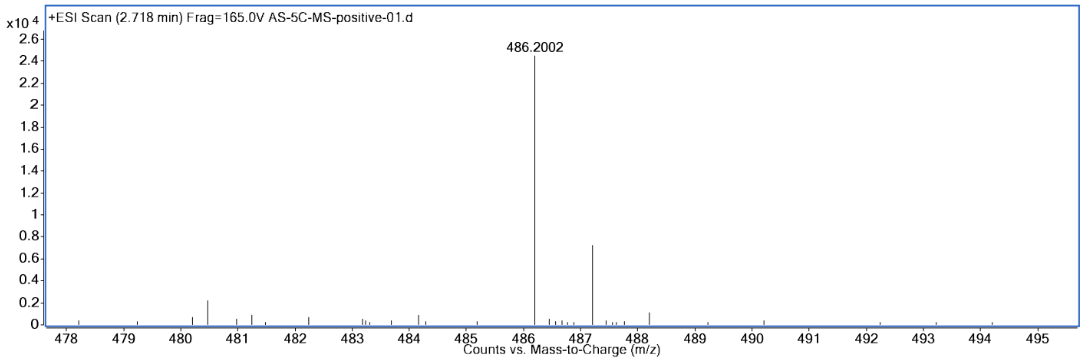

Figure S18 HRESIMS spectrum of compound **2**

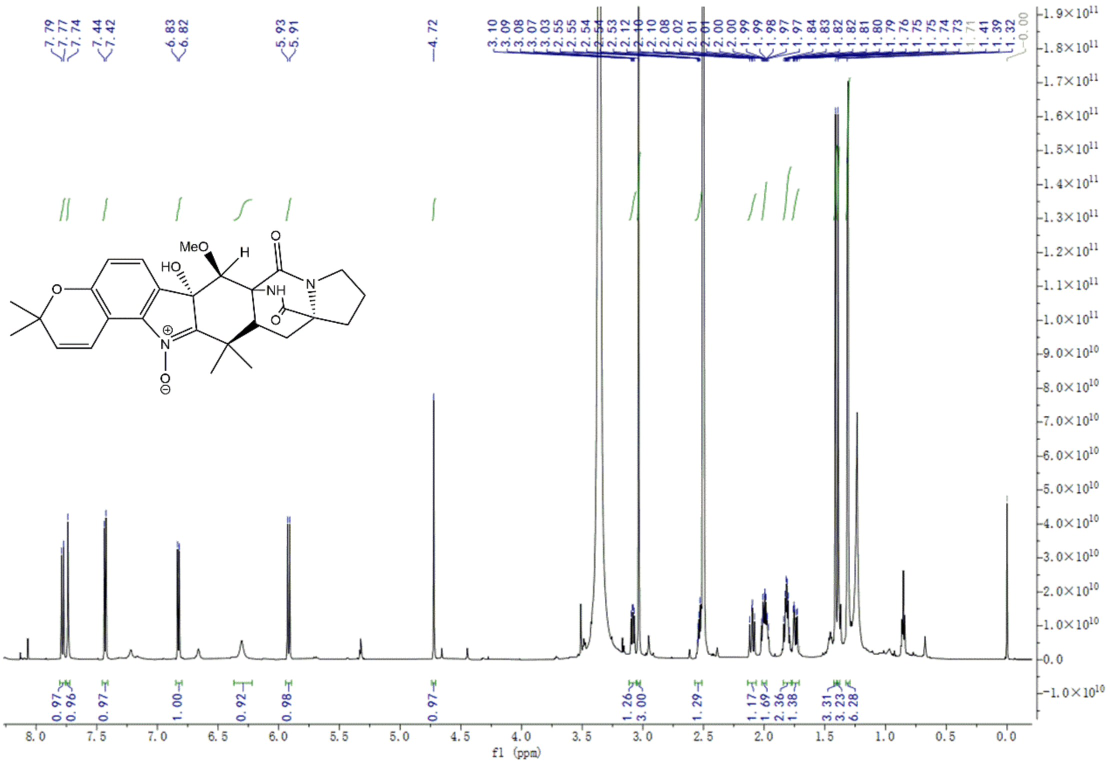

Figure S19 ^{1}H NMR spectrum (600 MHz) of compound **3** in DMSO-*d*6

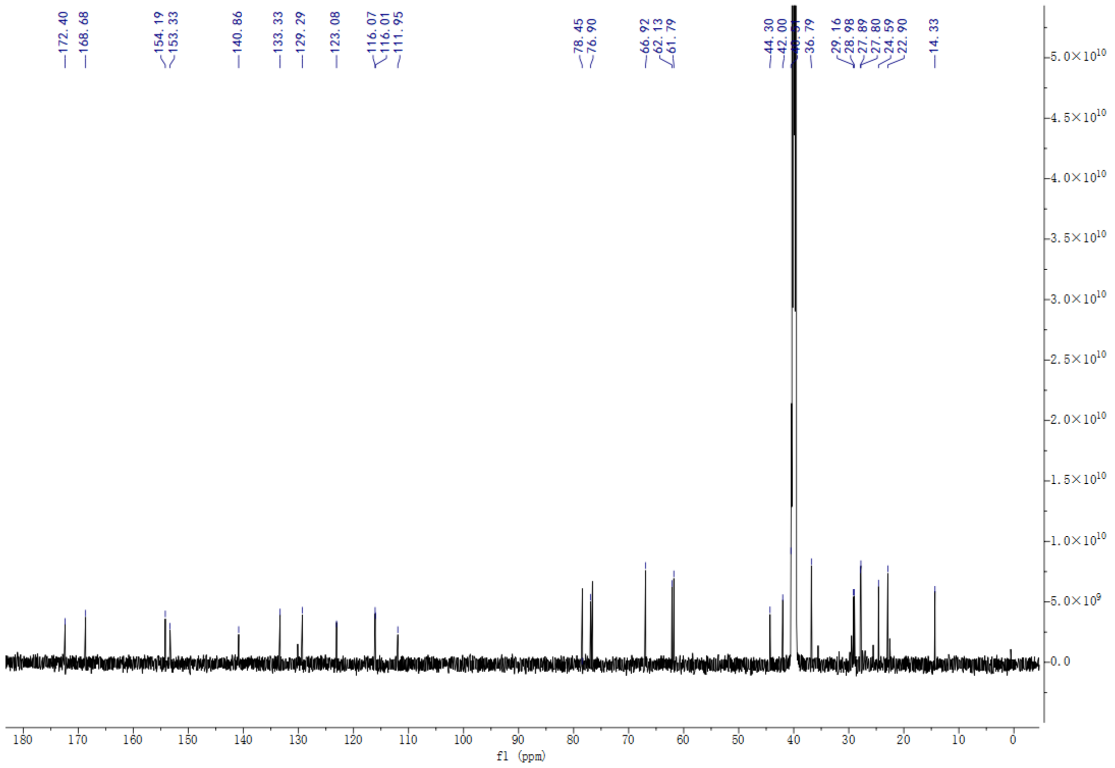

Figure S20 ^{13}C NMR spectrum (150 MHz) of compound **3** in DMSO -*d*6

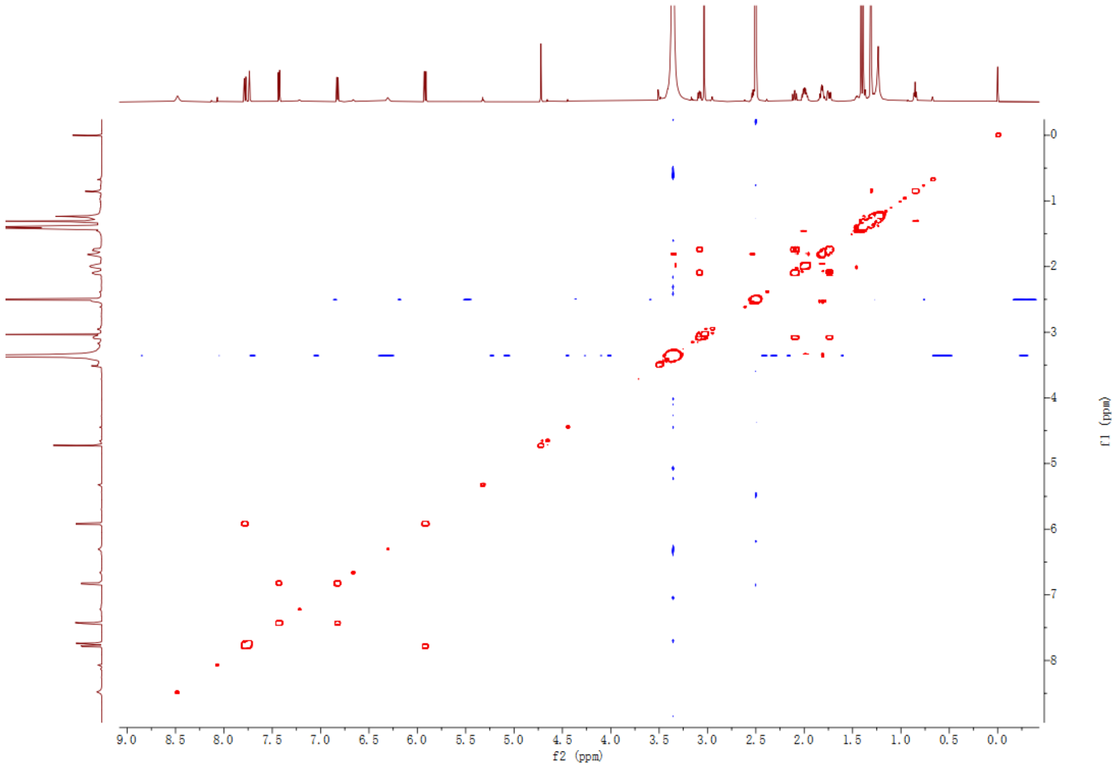

Figure S21 ^{1}H-^{1}H COSY spectrum of compound **3** in DMSO -*d*6

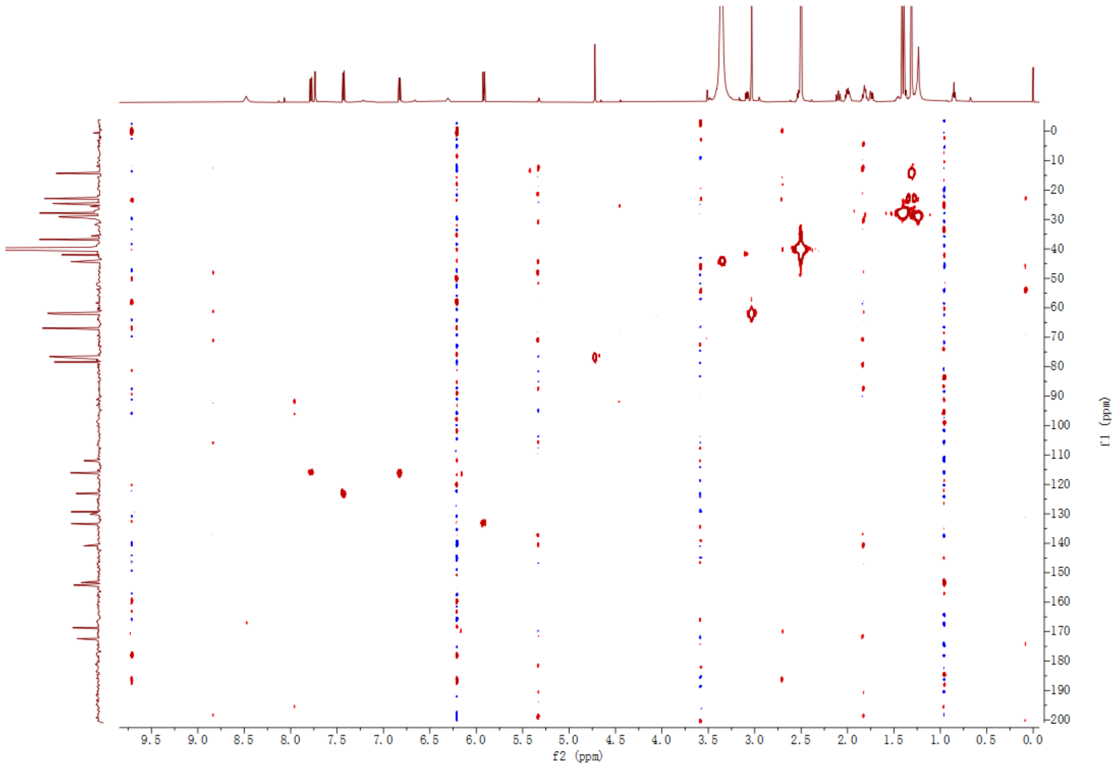

Figure S22 HSQC spectrum of compound **3** in DMSO -*d*6

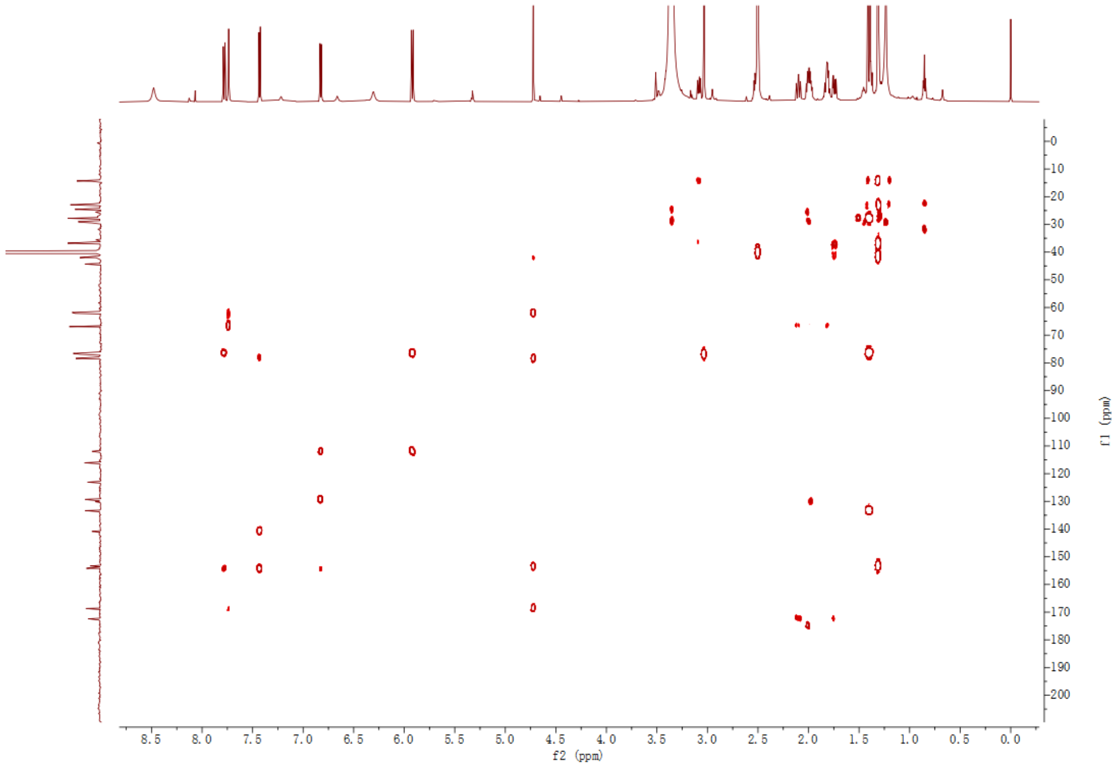

Figure S23 HMBC spectrum of compound **3** in DMSO -*d*6

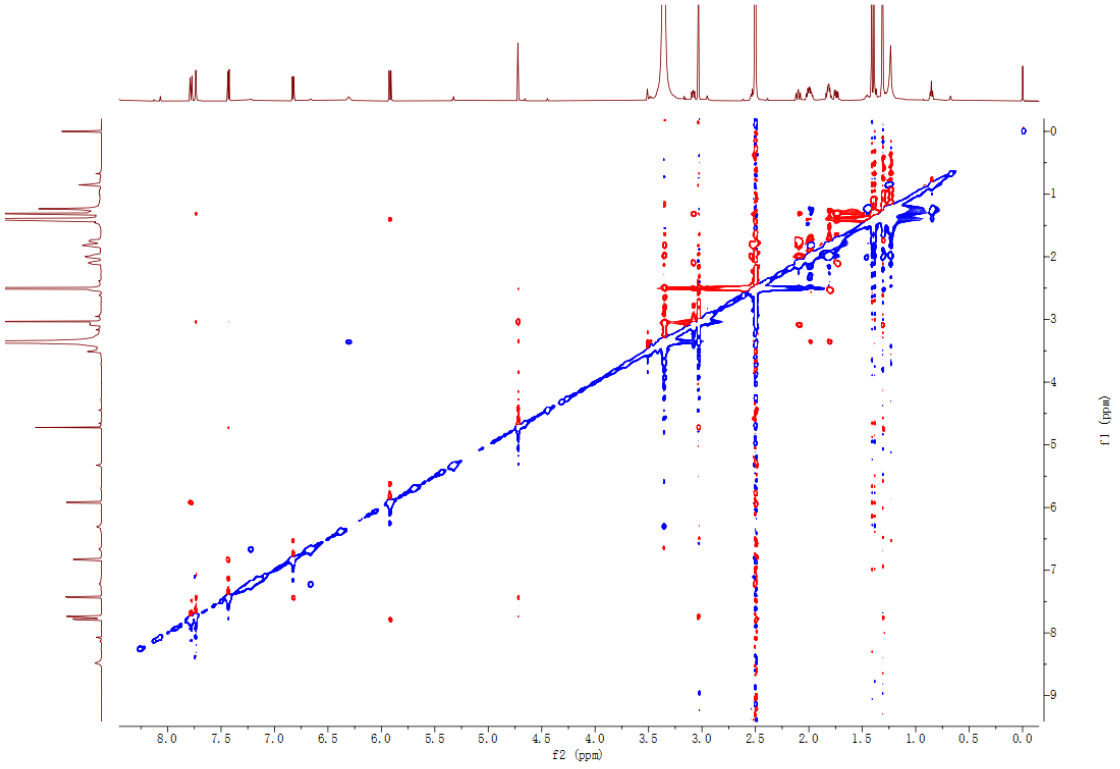

Figure S24 ROESY spectrum of compound **3** in DMSO -*d*6

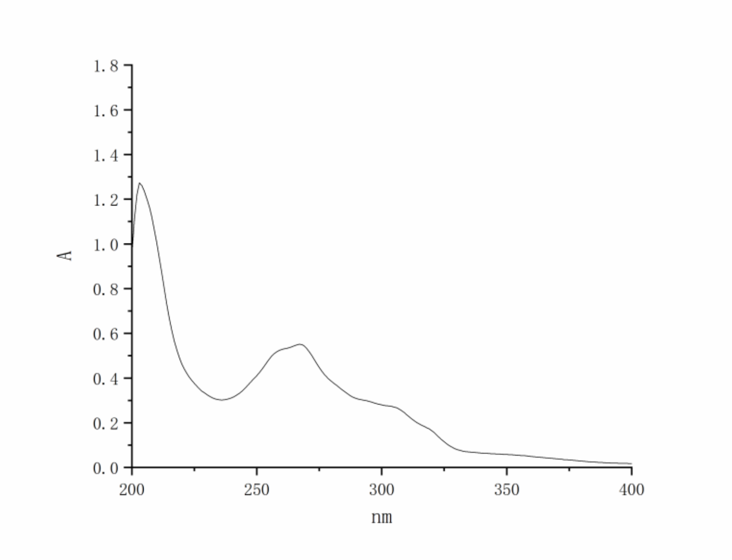

Figure S25 UV spectrum of compound **3** in MeOH

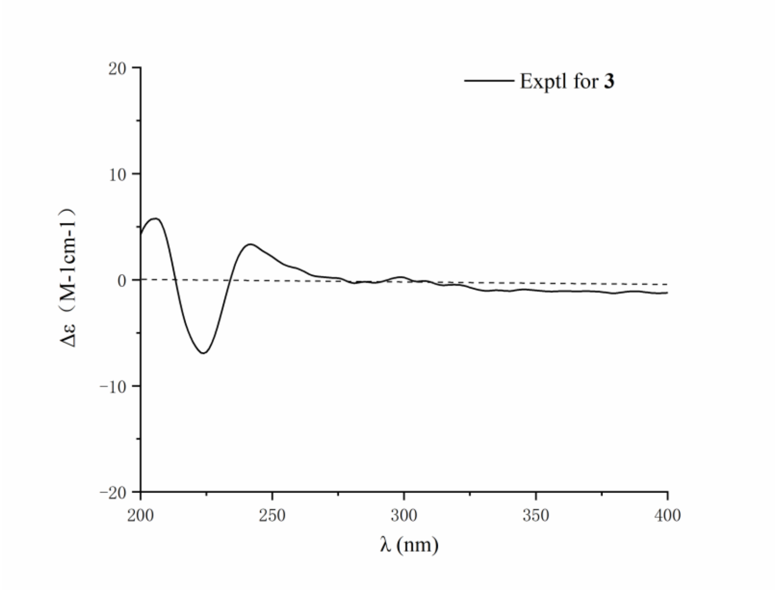

Figure S26 ECD spectrum of compound **3** in MeOH

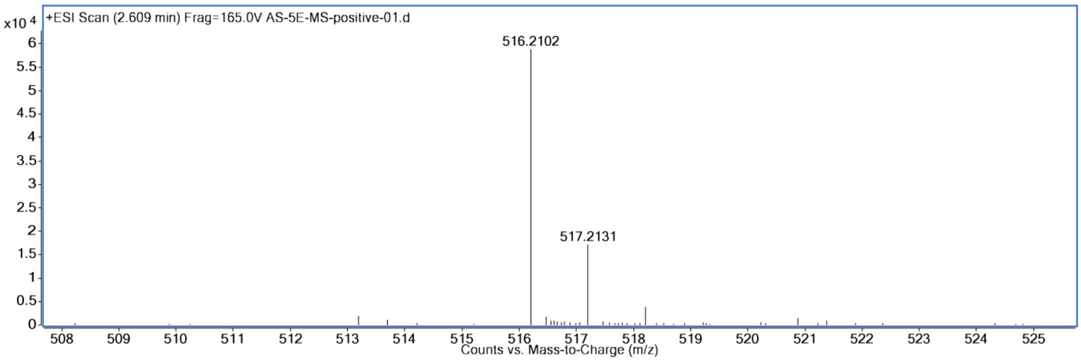

Figure S27 HRESIMS spectrum of compound **3**

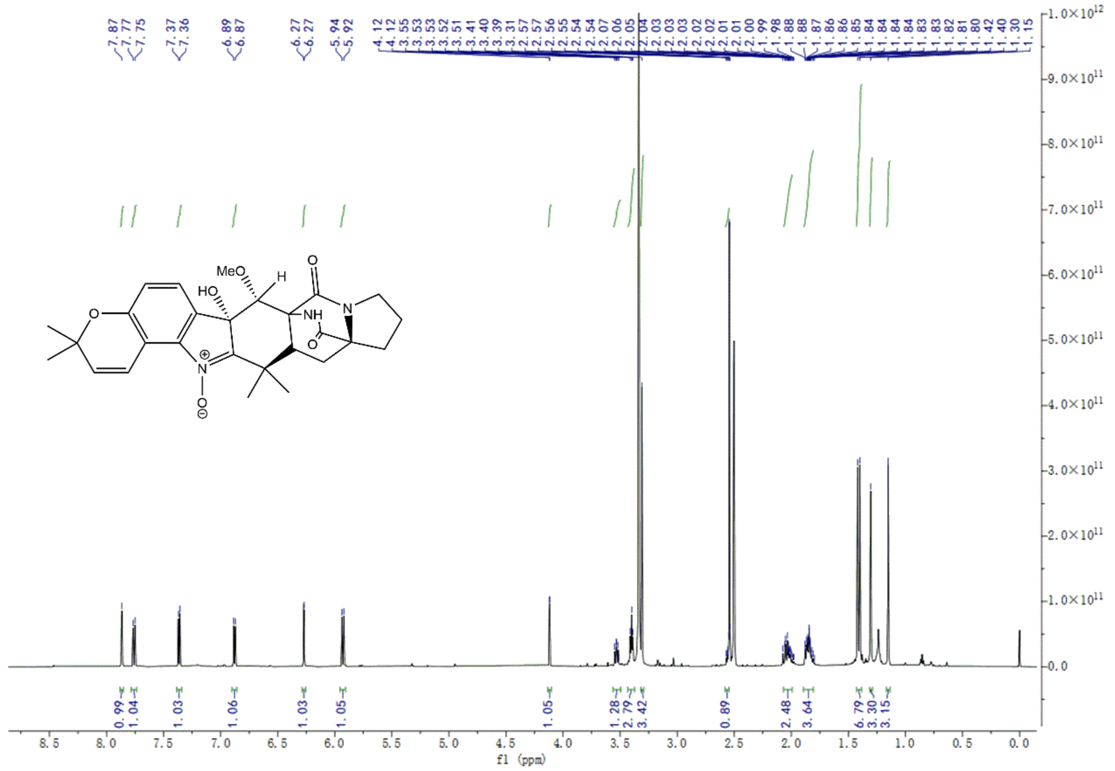

Figure S28 ^{1}H NMR spectrum (600 MHz) of compound **4** in DMSO-d_6

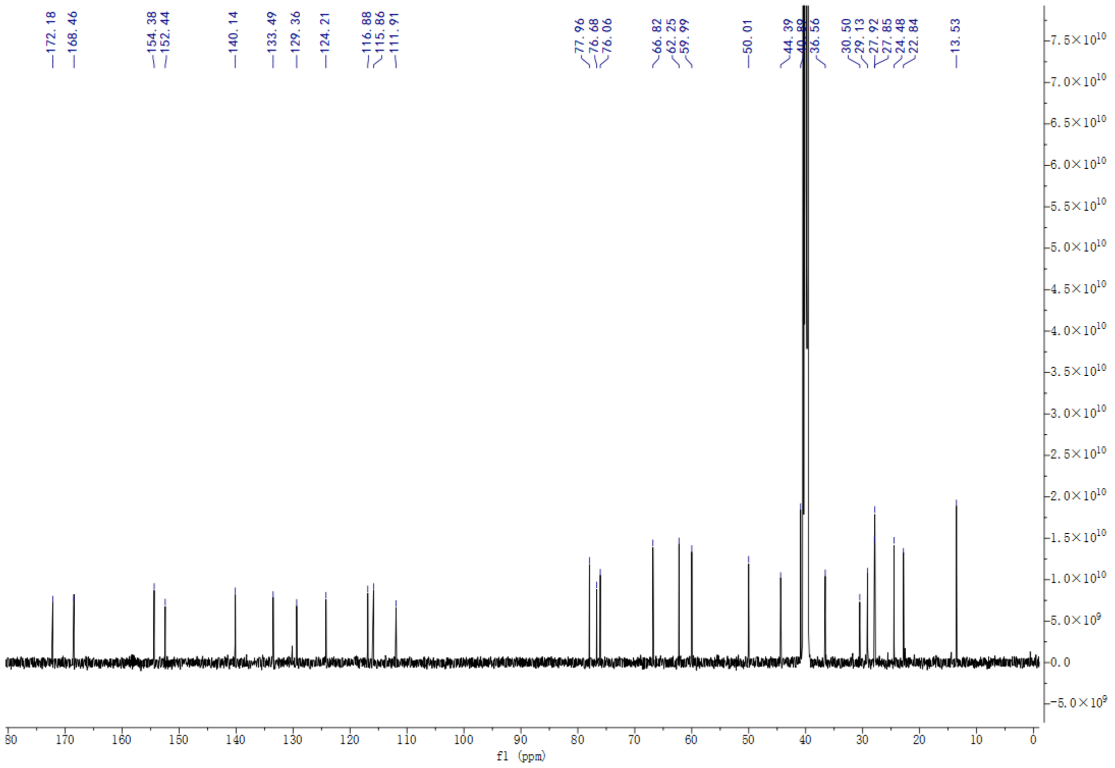

Figure S29 ^{13}C NMR spectrum (150 MHz) of compound **4** in DMSO -d_6

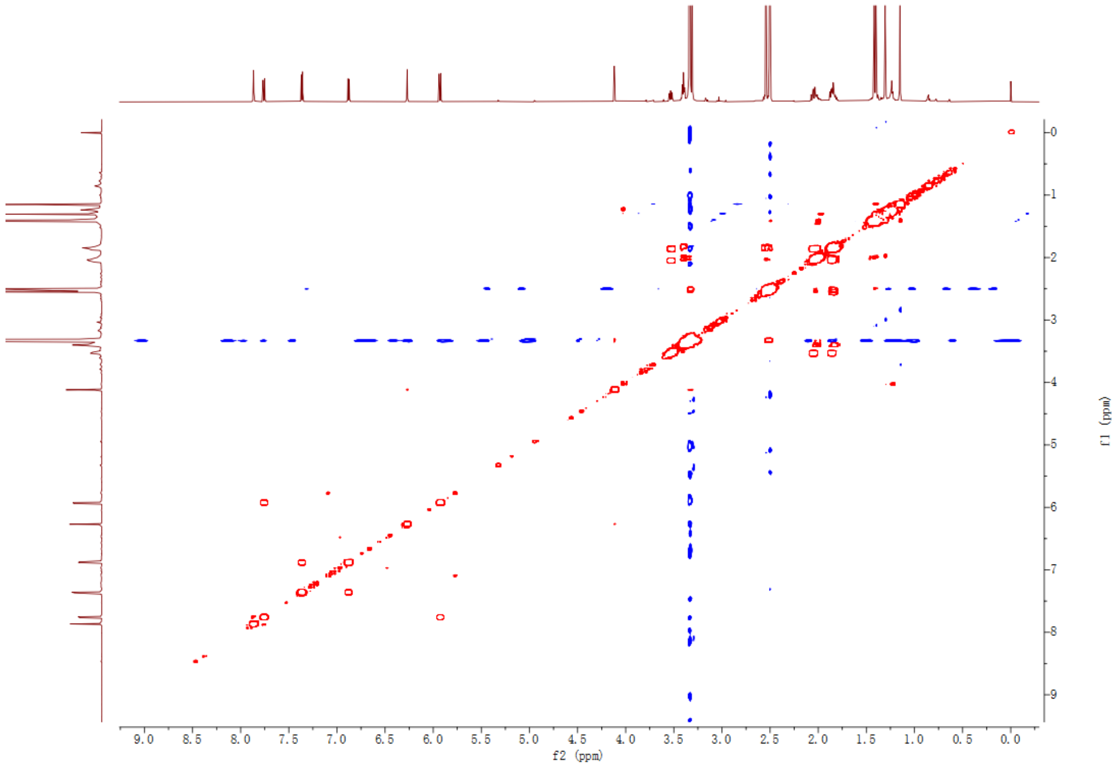

Figure S30 ^{1}H-^{1}H COSY spectrum of compound **4** in DMSO -d_6

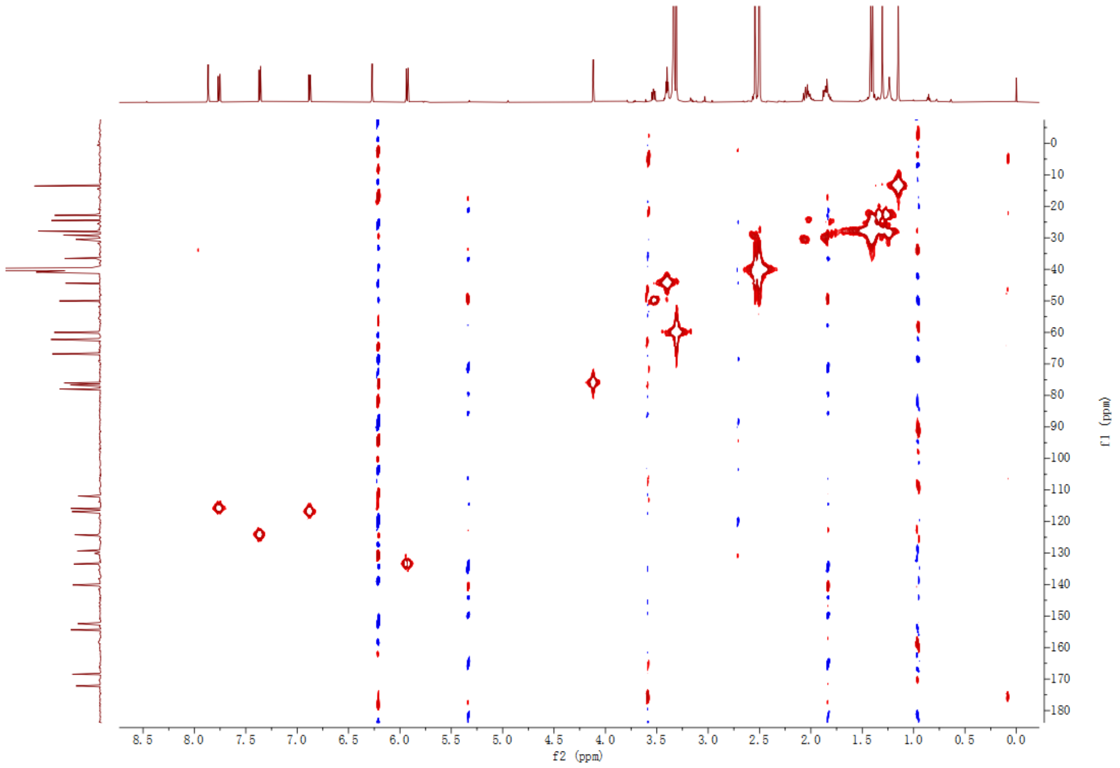

Figure S31 HSQC spectrum of compound **4** in DMSO -d_6

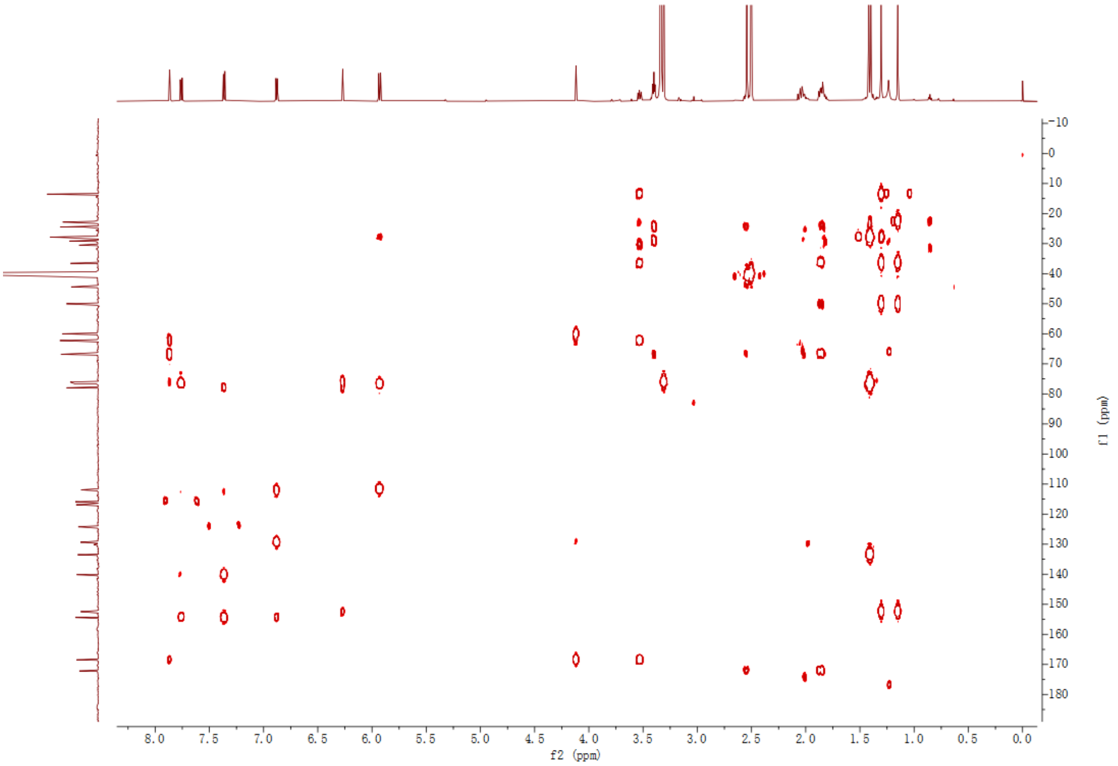

Figure S32 HMBC spectrum of compound **4** in DMSO -d_6

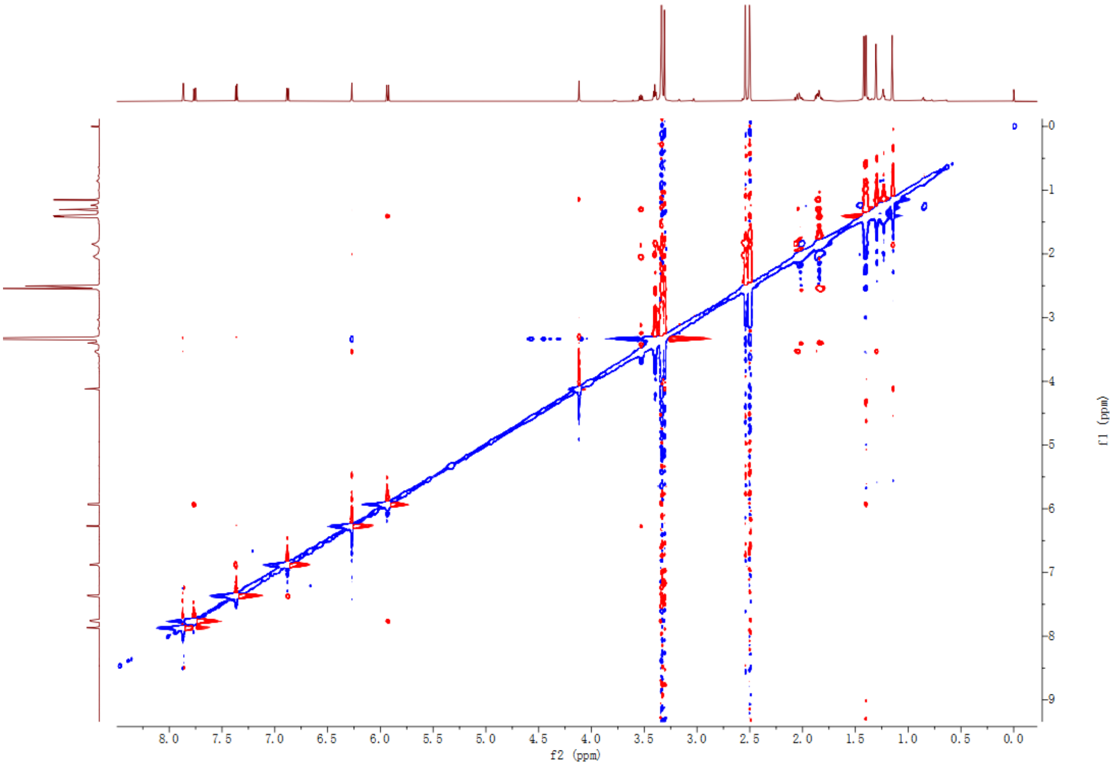

Figure S33 ROESY spectrum of compound **4** in DMSO -d_6

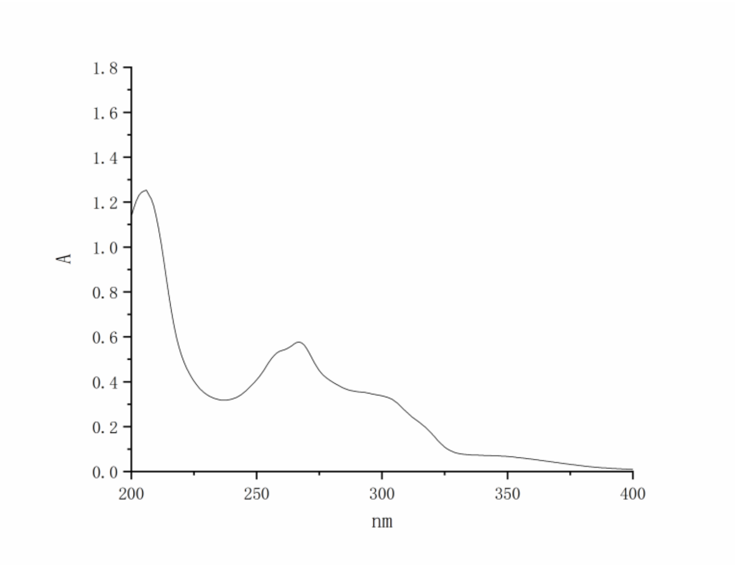

Figure S34 UV spectrum of compound **4** in MeOH

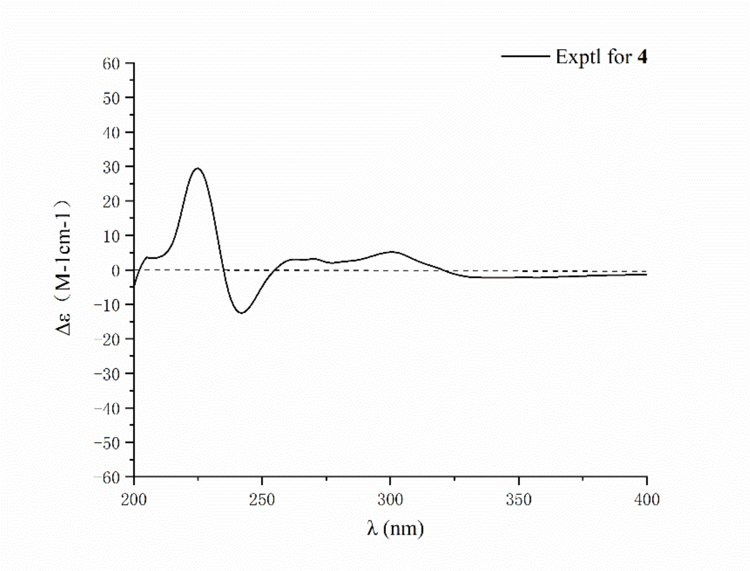

Figure S35 ECD spectrum of compound **4** in MeOH

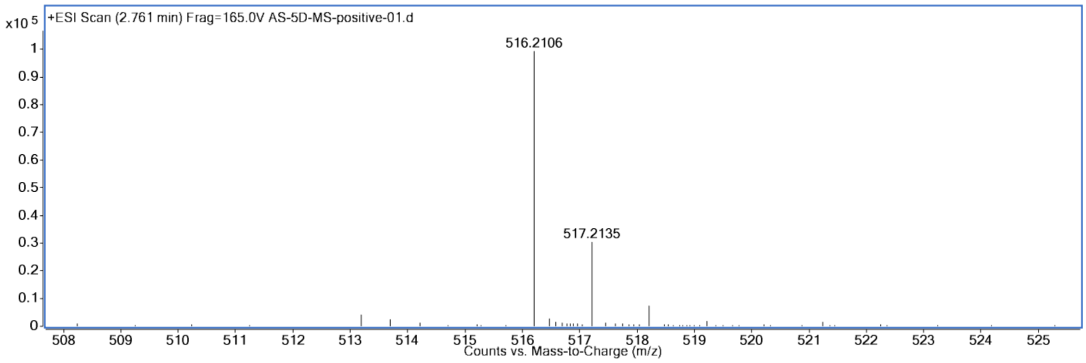

Figure S36 HRESIMS spectrum of compound **4**

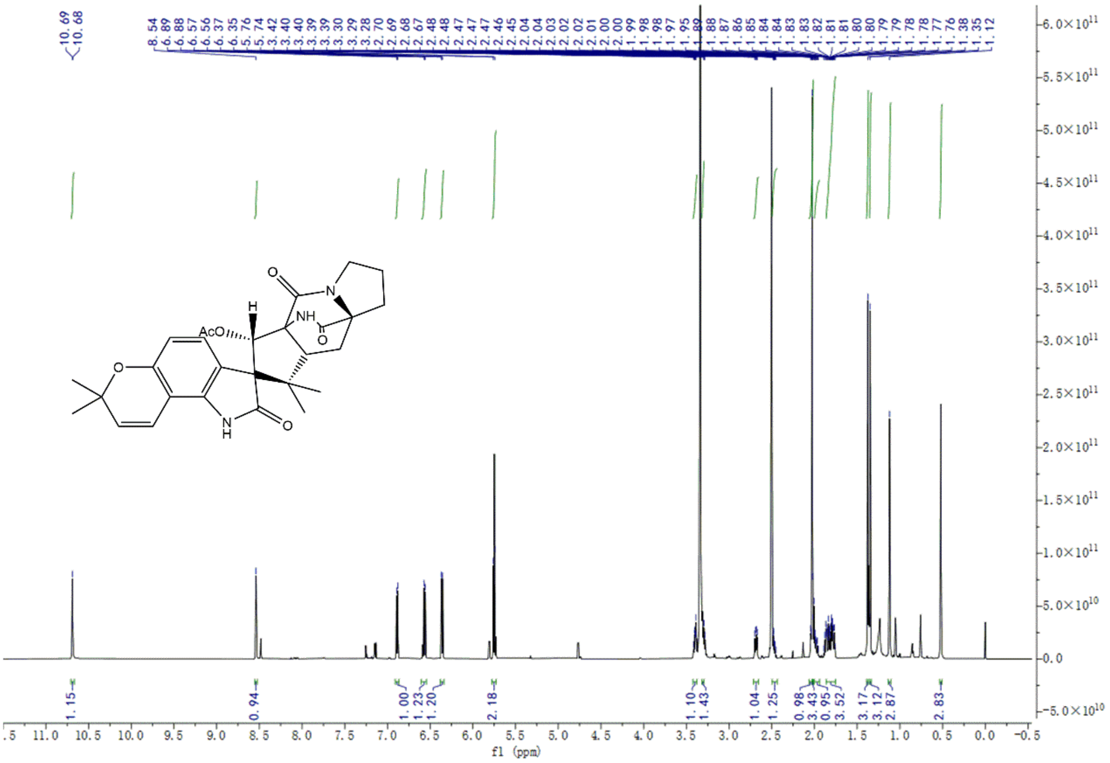

Figure S37 ^{1}H NMR spectrum (600 MHz) of compound **5** in DMSO-*d*6

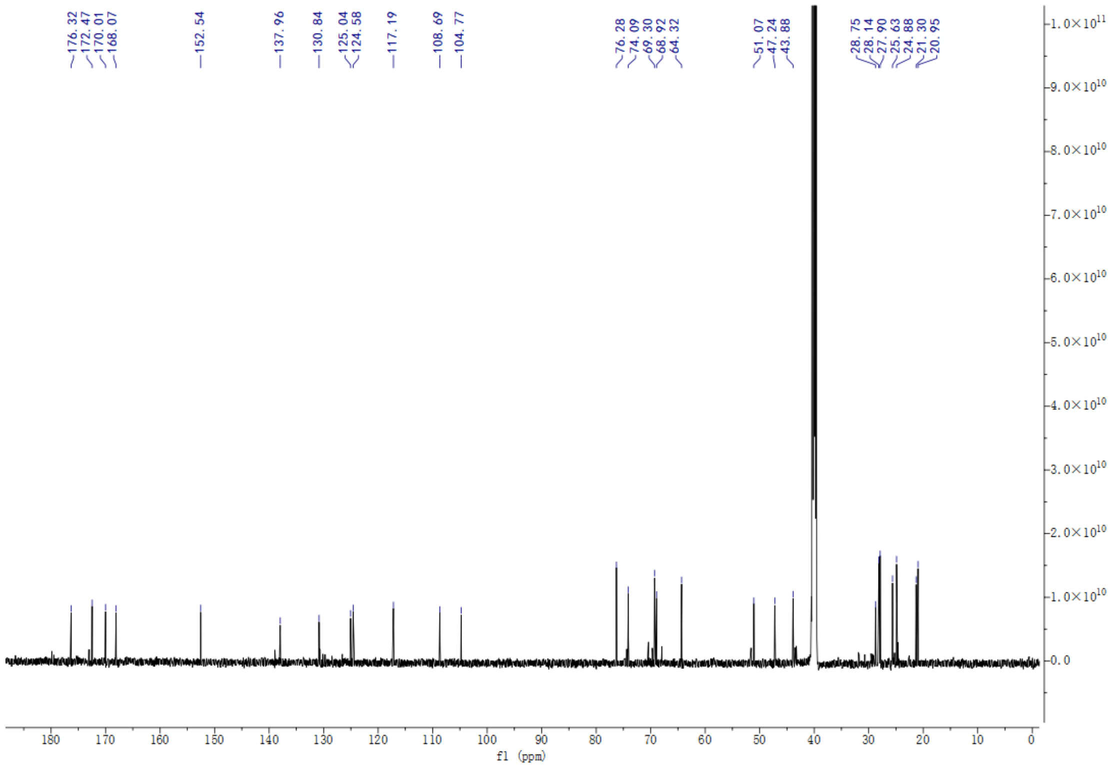

Figure S38 ^{13}C NMR spectrum (150 MHz) of compound **5** in DMSO -*d*6

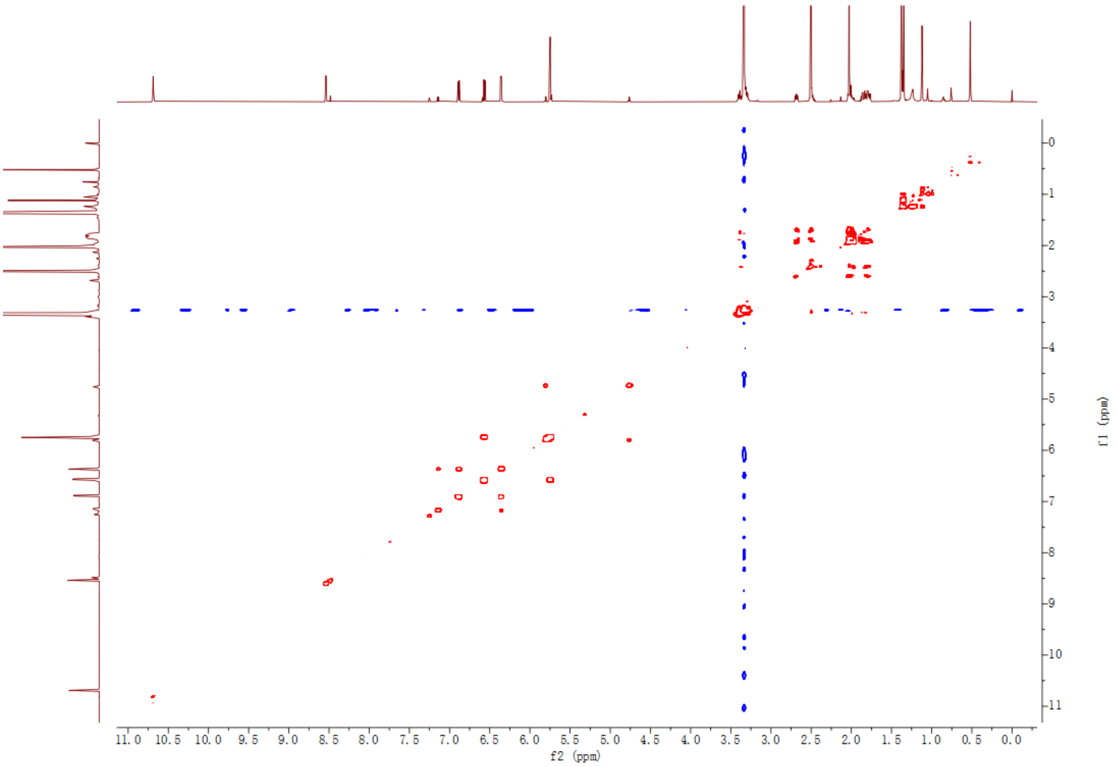

Figure S39 ^{1}H-^{1}H COSY spectrum of compound **5** in DMSO -*d*6

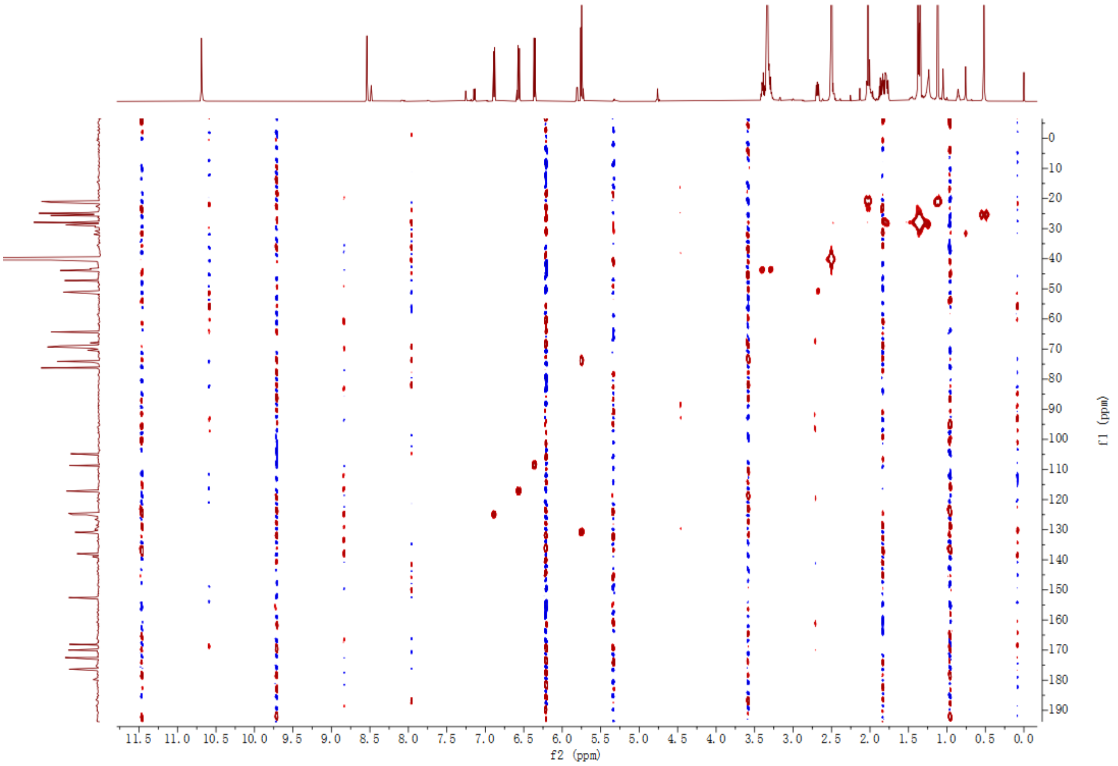

Figure S40 HSQC spectrum of compound **5** in DMSO -*d*6

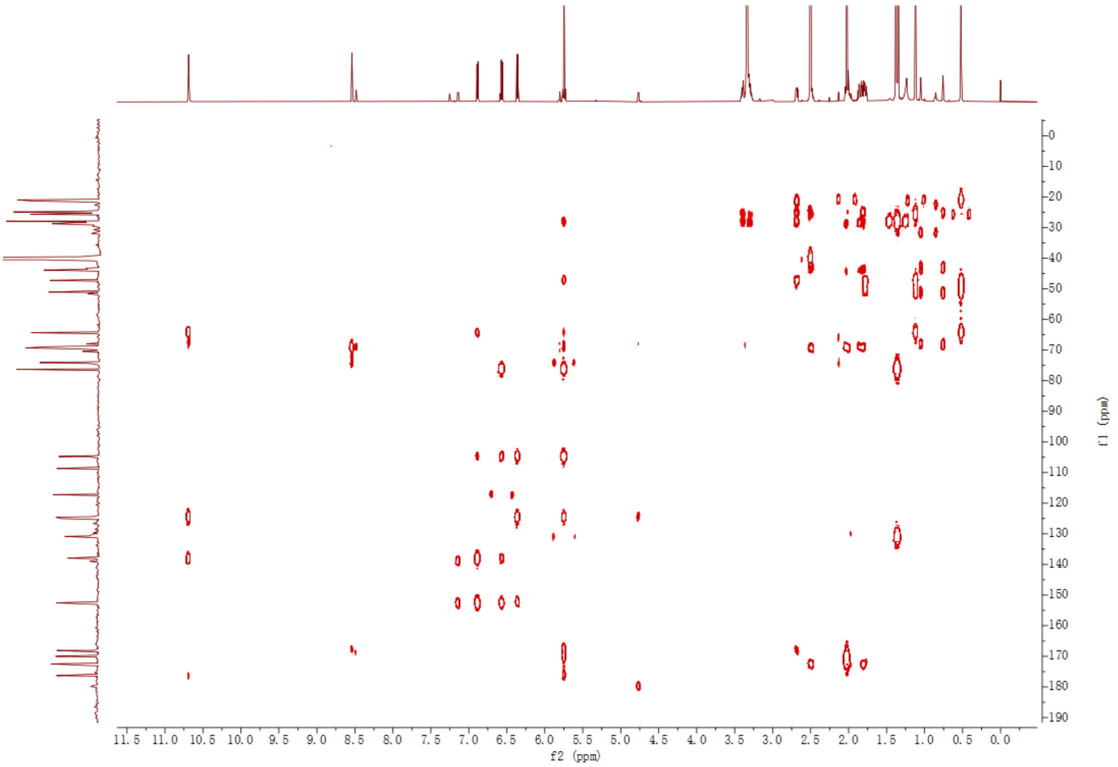

Figure S41 HMBC spectrum of compound **5** in DMSO -*d*6

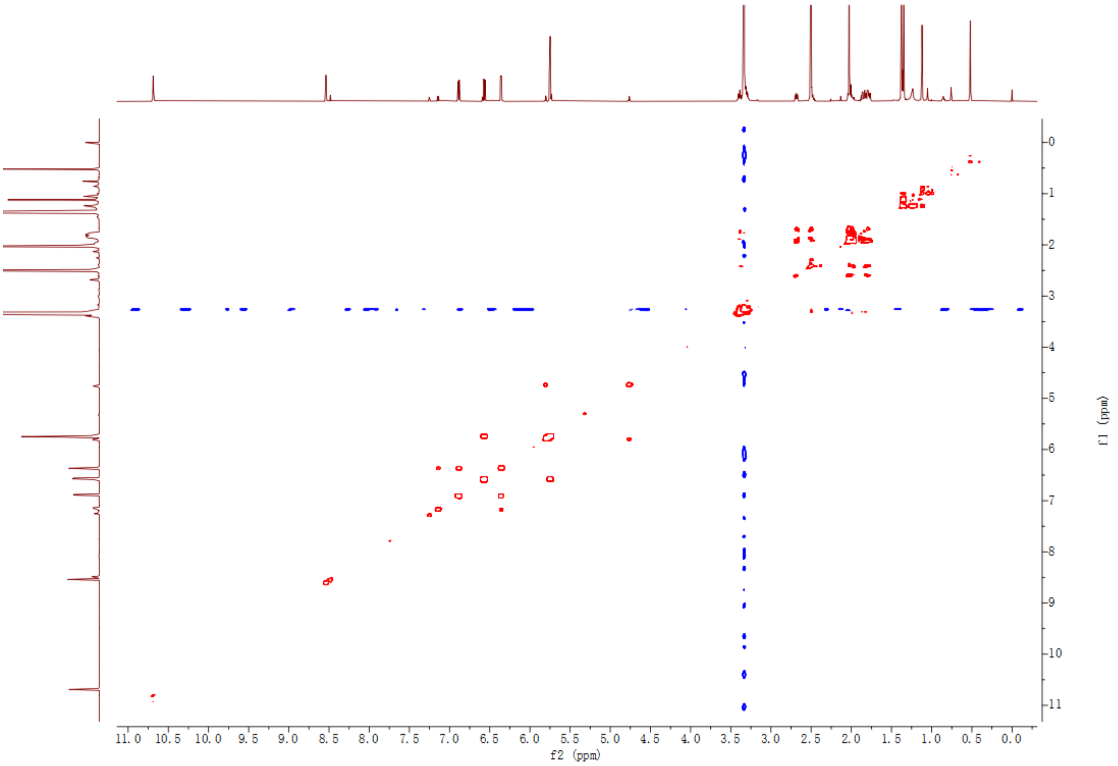

Figure S42 ROESY spectrum of compound **5** in DMSO -*d*6

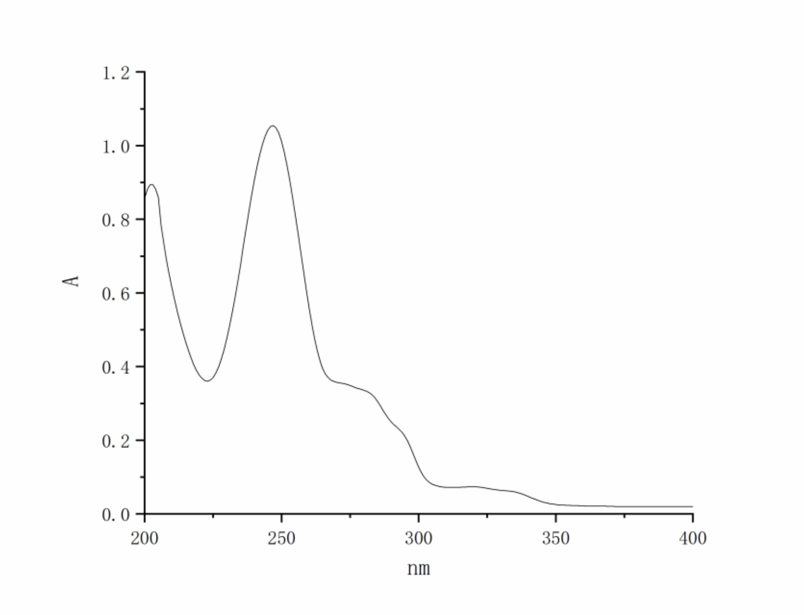

Figure S43 UV spectrum of compound **5** in MeOH

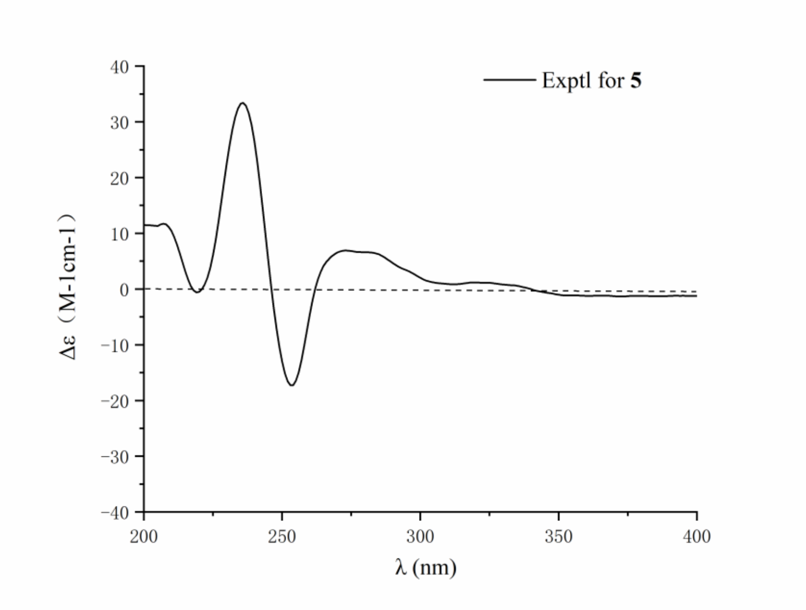

Figure S44 ECD spectrum of compound **5** in MeOH

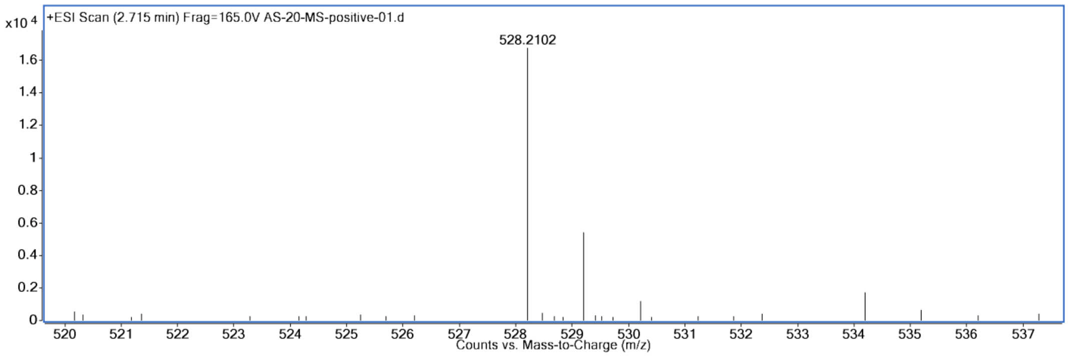

Figure S45 HRESIMS spectrum of compound **5**

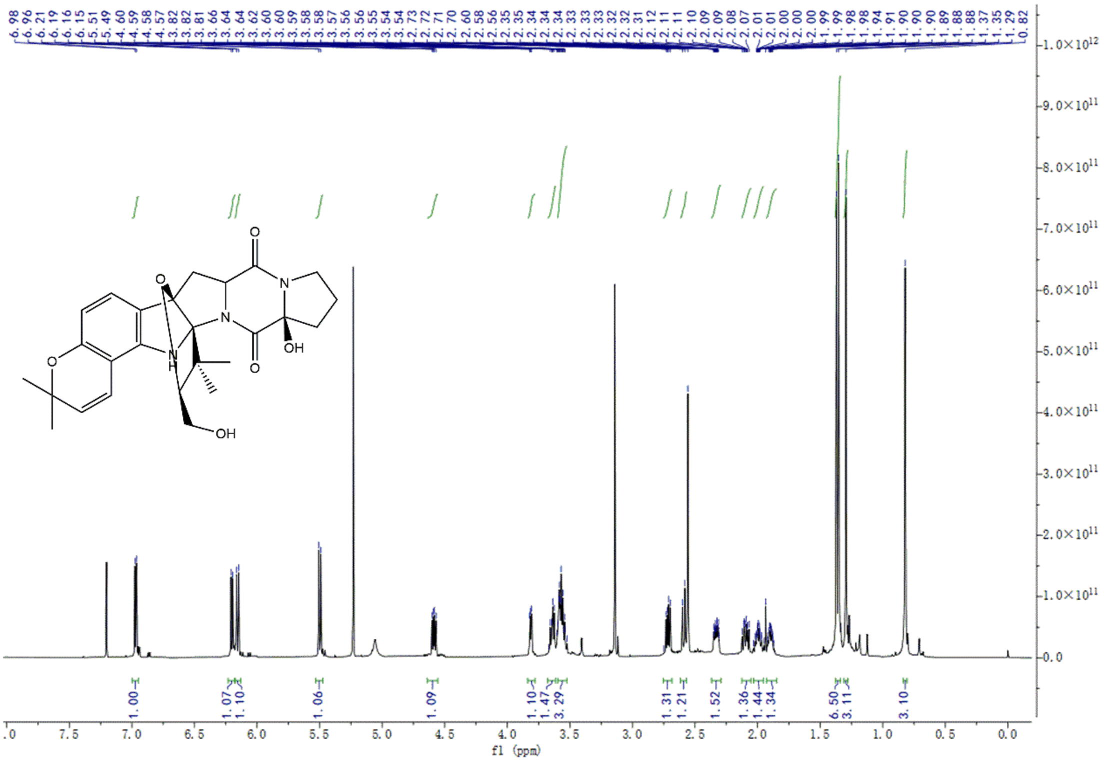

Figure S46 ^{1}H NMR spectrum (600 MHz) of compound **6** in $CDCl_3$

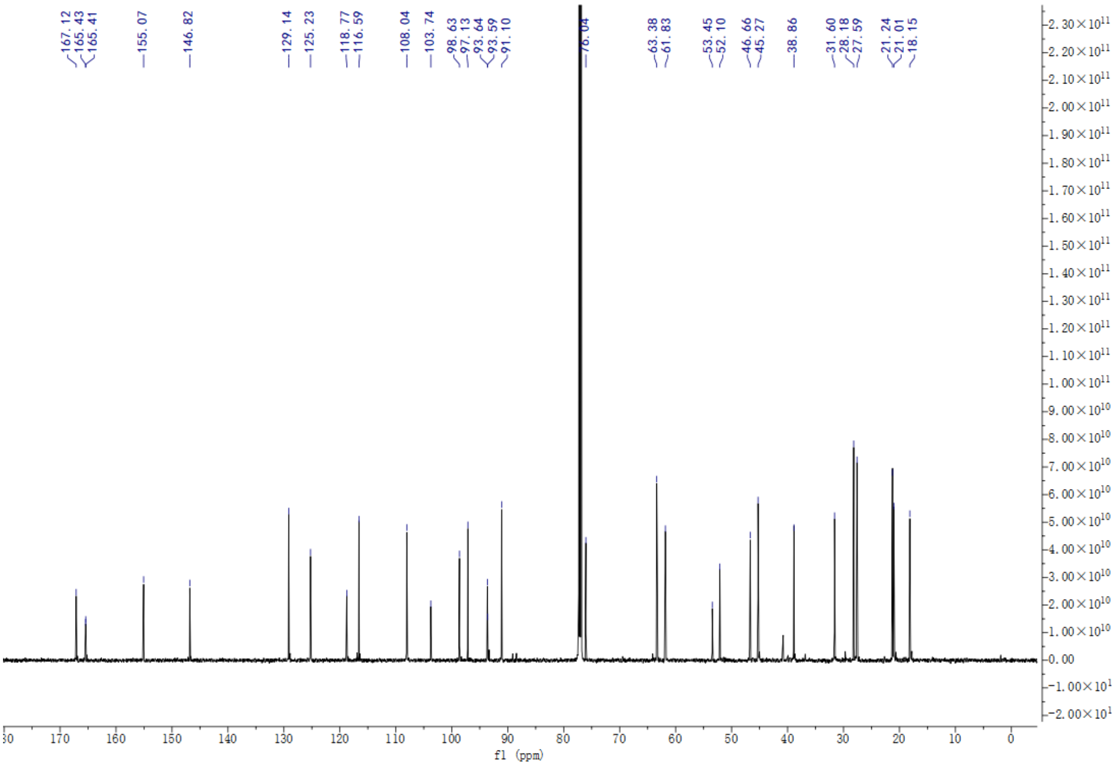

Figure S47 ^{13}C NMR spectrum (150 MHz) of compound **6** in $CDCl_3$

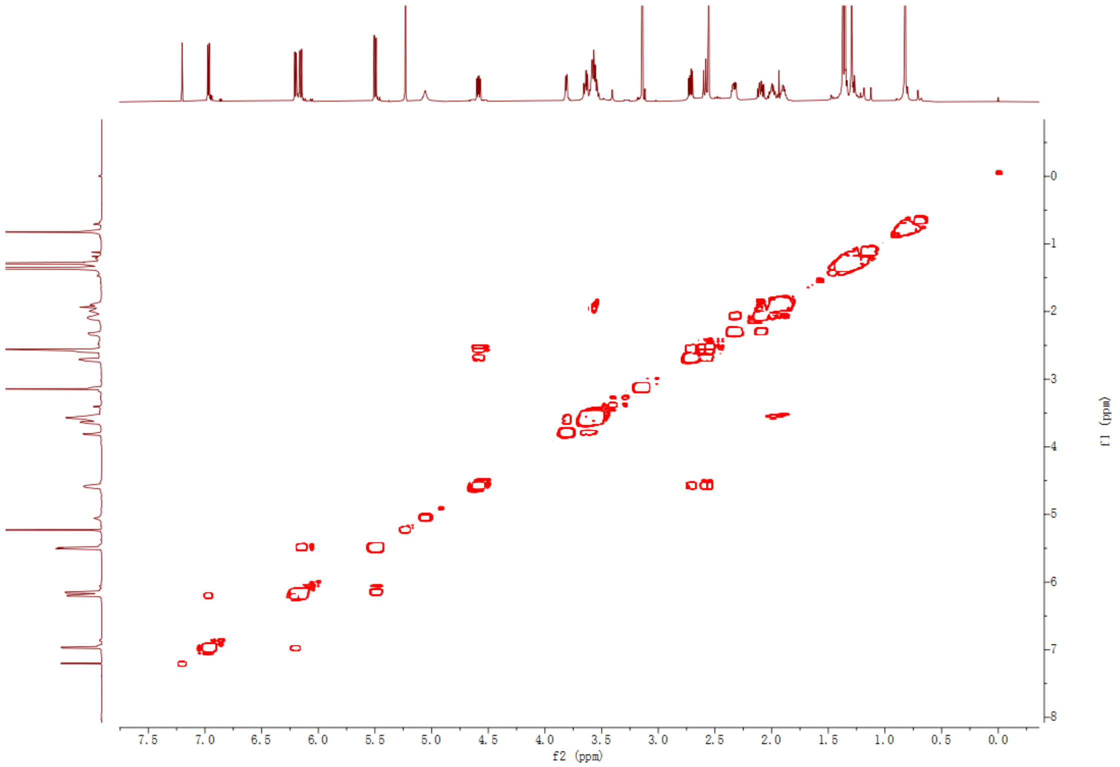

Figure S48 ^{1}H-^{1}H COSY spectrum of compound **6** in $CDCl_3$

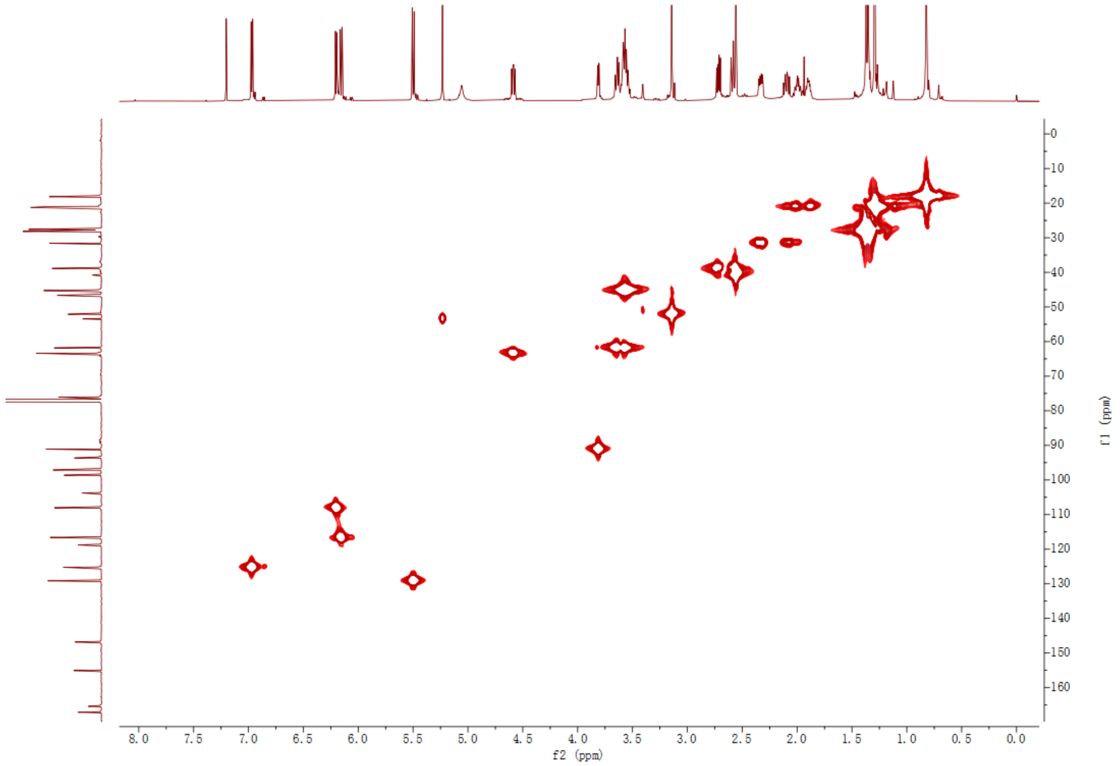

Figure S49 HSQC spectrum of compound **6** in $CDCl_3$

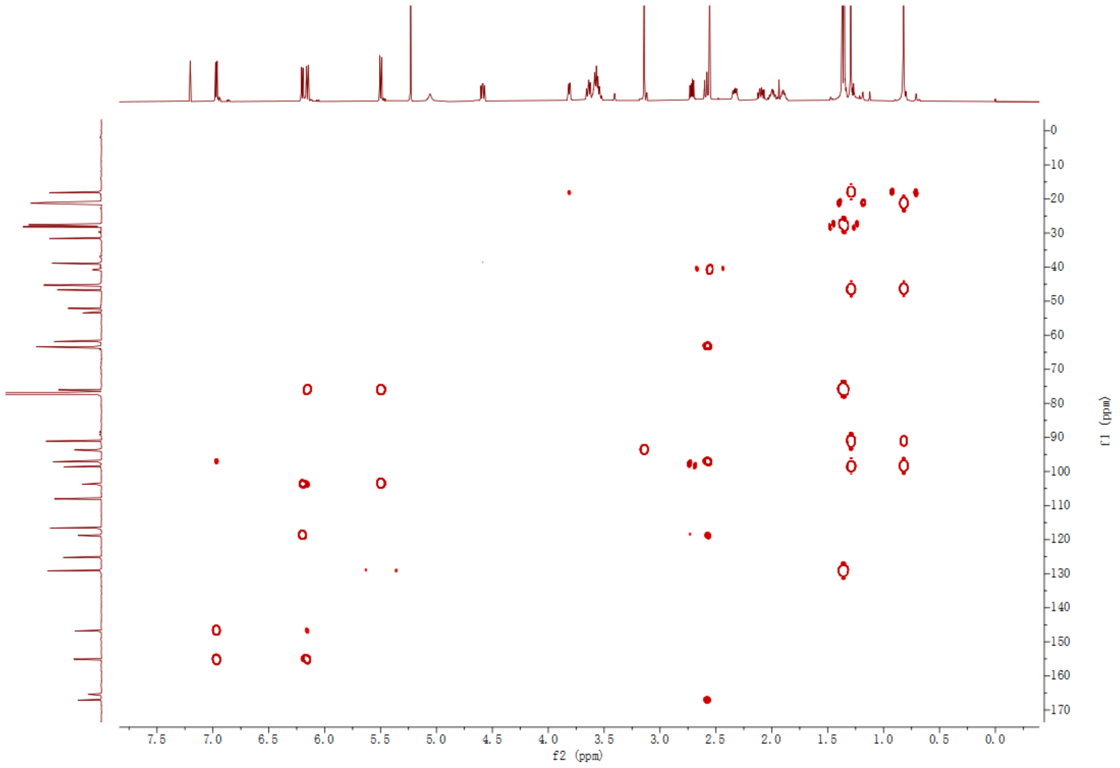

Figure S50 HMBC spectrum of compound **6** in $CDCl_3$

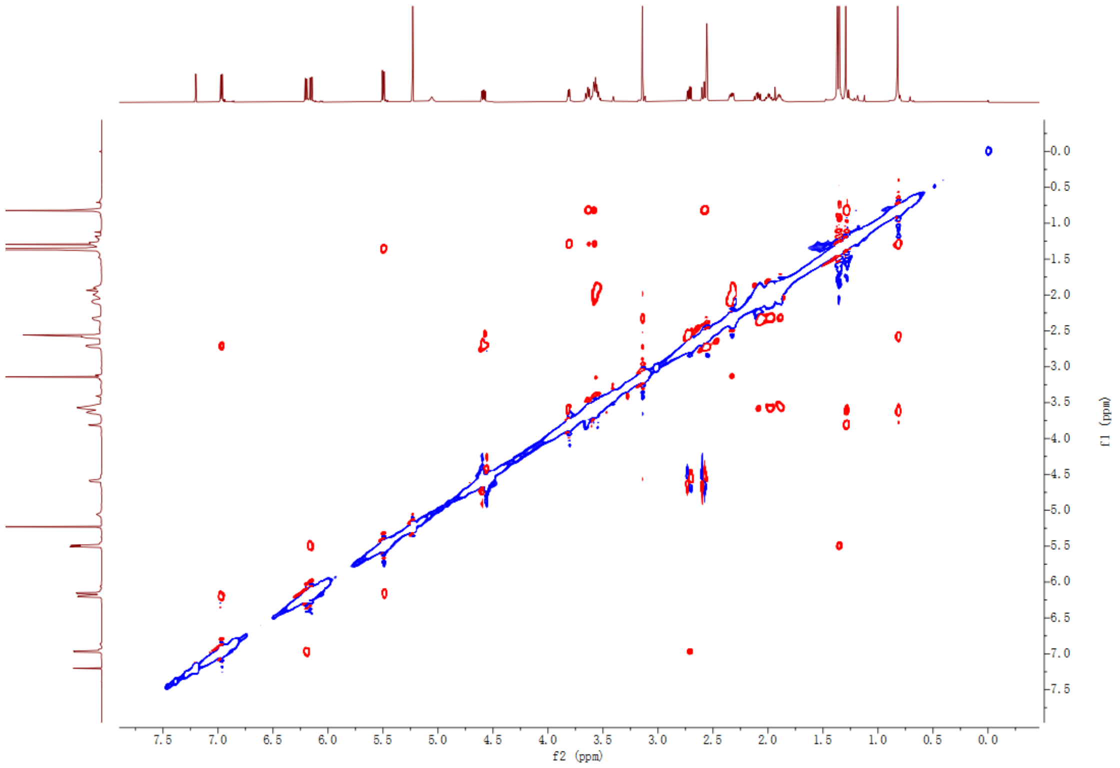

Figure S51 ROESY spectrum of compound **6** in $CDCl_3$

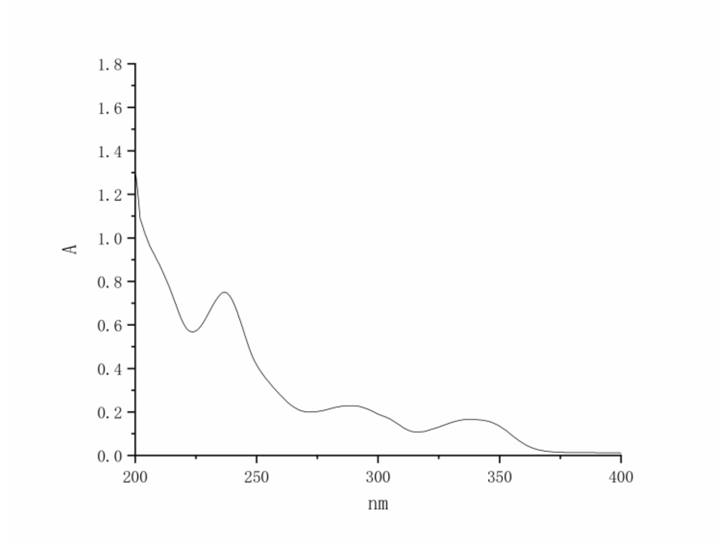

Figure S52 UV spectrum of compound **6** in MeOH

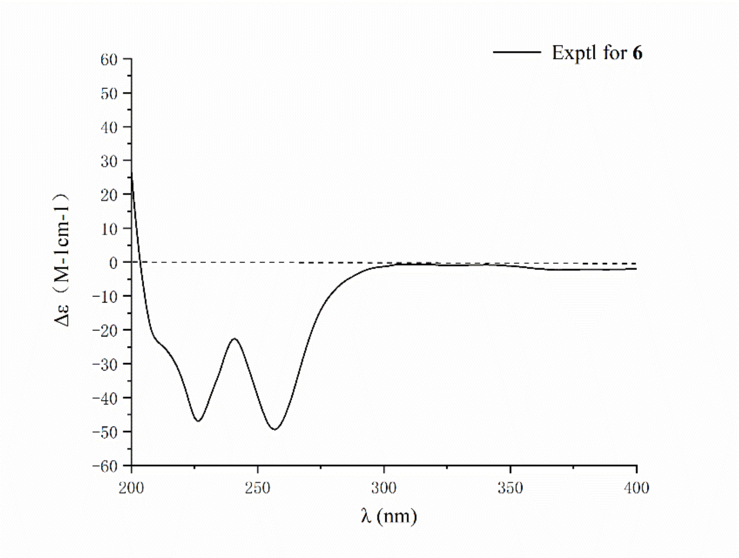

Figure S53 ECD spectrum of compound **6** in MeOH

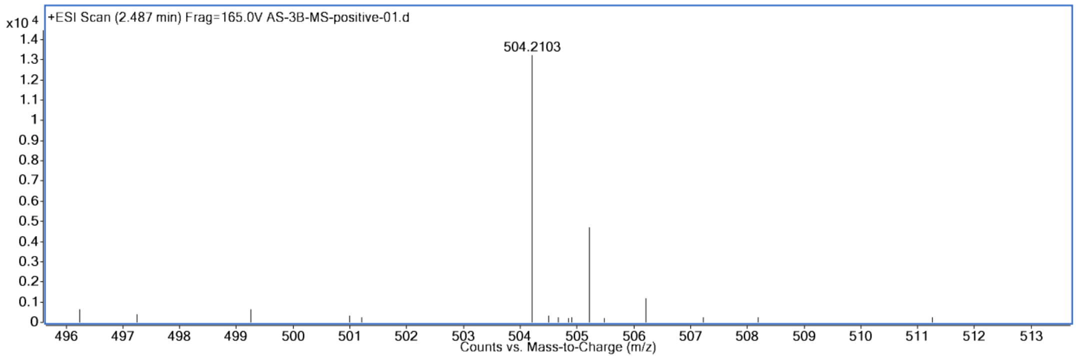

Figure S54 HRESIMS spectrum of compound **6**

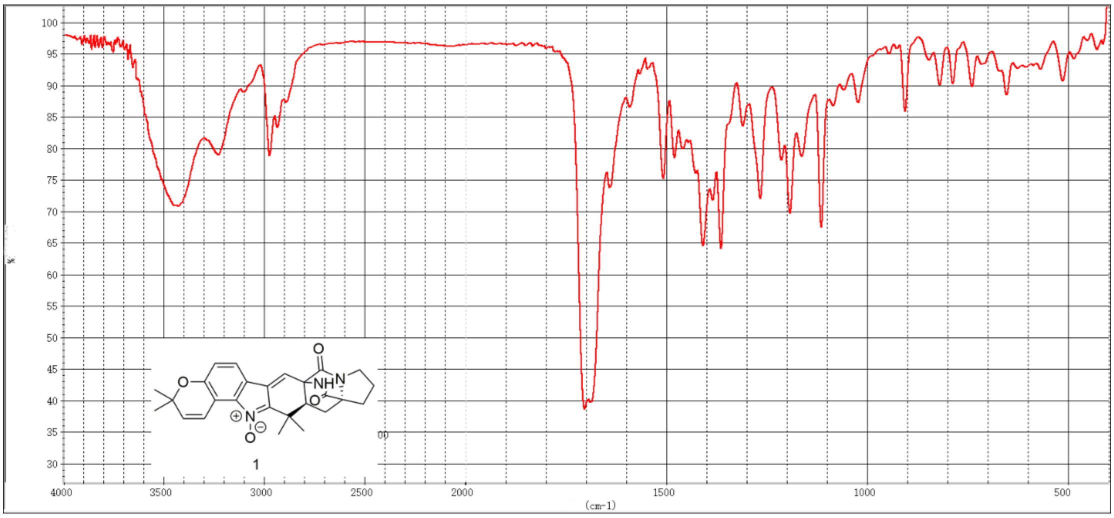

Figure S55 IR spectrum of compound **1**

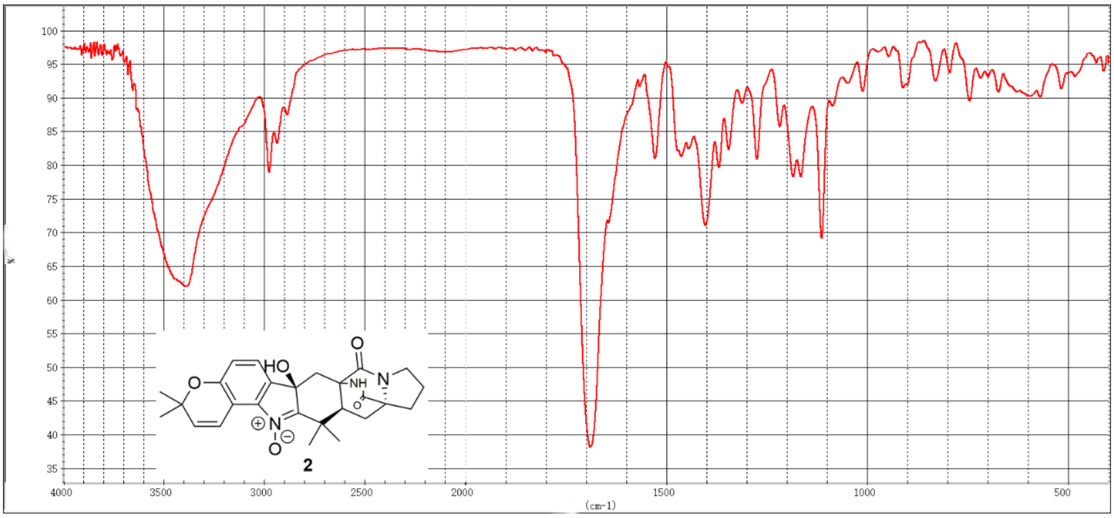

Figure S56 IR spectrum of compound **2**

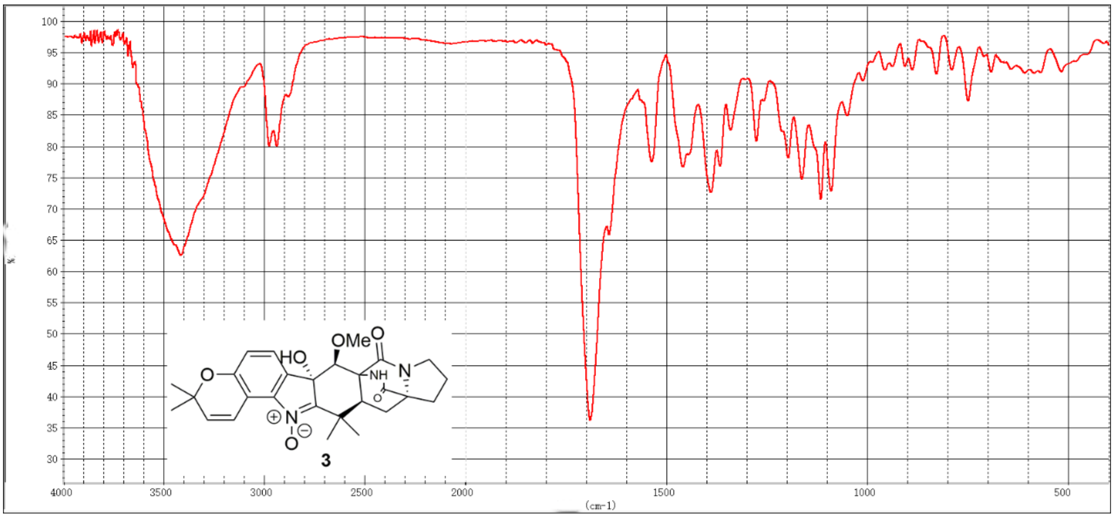

Figure S57 IR spectrum of compound **3**

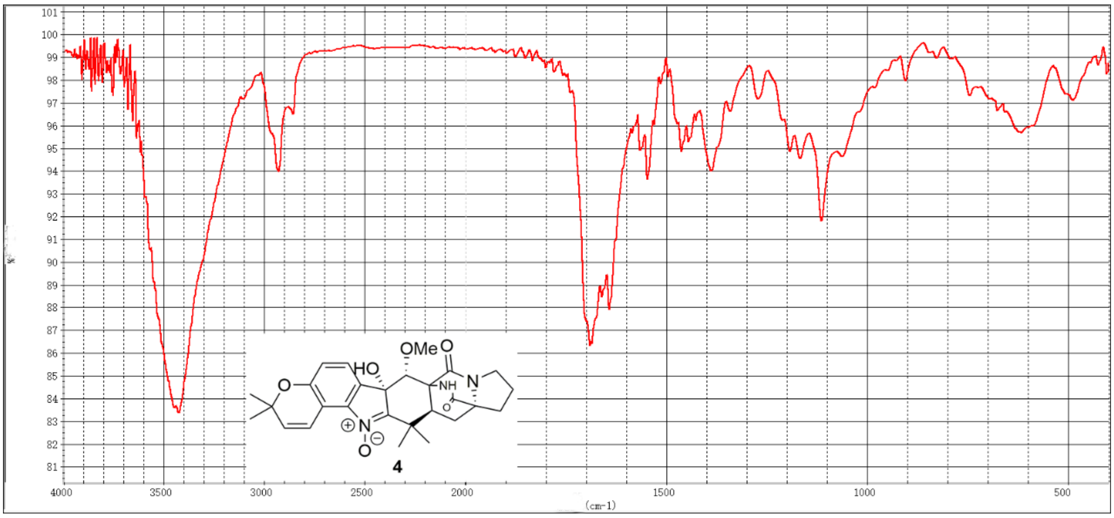

Figure S58 IR spectrum of compound **4**

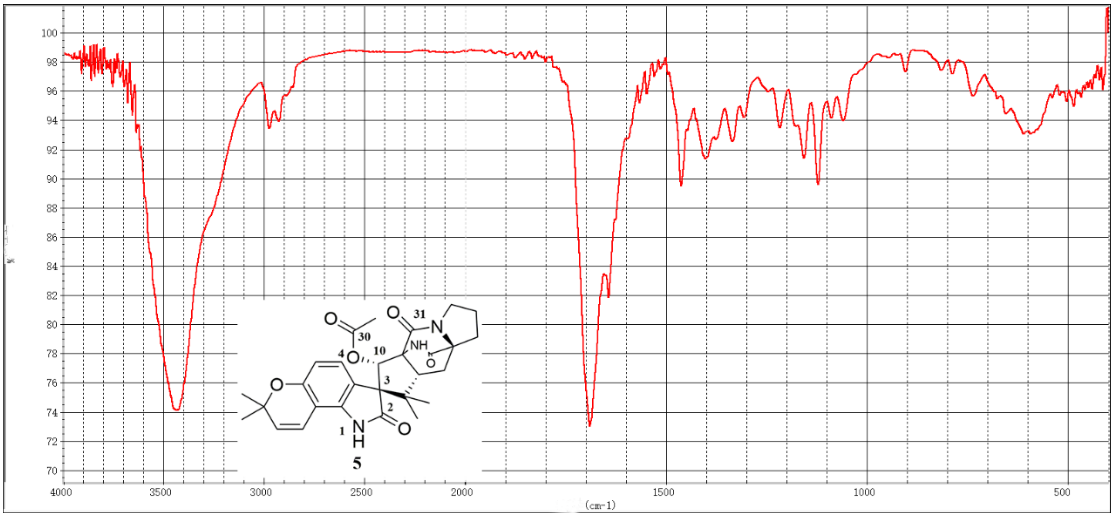

Figure S59 IR spectrum of compound **5**

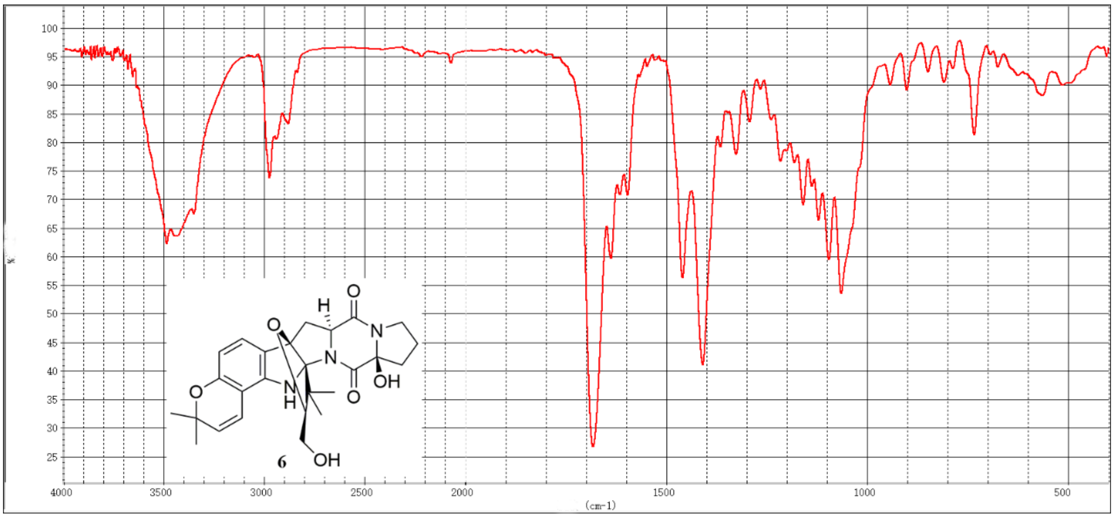

Figure S60 IR spectrum of compound **6**

ECD Calculation

Monte Carlo conformational searches were carried out by means of the Spartan's 14 software using Merck Molecular Force Field (MMFF). The conformers with Boltzmann-population of over 5% were chosen for ECD calculations, and then the conformers were initially optimized at B3LYP/6-31g level in gas. The theoretical calculation of ECD was conducted in MeOH using Time-dependent Density functional theory (TD-DFT) at the B3LYP/6-31+g (d, p) level for all conformers of compounds **1-6**. Rotatory strengths for a total of 30 excited states were calculated. ECD spectra were generated using the program SpecDis 1.6 (University of Würzburg, Würzburg, Germany) and GraphPad Prism 5 (University of California San Diego, USA) from dipole-length rotational strengths by applying Gaussian band shapes with sigma = 0.3 eV.

Antimicrobial Assay

The pathogenic fungus was activated on a PDA medium plate and cultured at 28 °C for 2-3 days. The pathogenic fungus-contianing agar was inoculated into a 250 mL Erlenmeyer flask containing 100 mL of PDB medium, culture it at 28 °C, 160 r/min for 2-3 days. The solvent DMSO was selected as a negative control and ketoconazole was dissolved into DMSO at the concentration of 10 mg/mL as a positive control. Besides, fungal suspension was used as a blank control. The tested compounds were dissolved into DMSO at the concentration of 10 mg/mL. 98 μL fungal suspension (10^4 mycelia fragments/ml) and 2 μL sample solution were added into the first row of the 96-well plate with 100 μL medium (peptone 10 g/L and glucose 40 g/L). 2 μL of blank control solution, positive control solution, and negative control solution were added to the last three columns. Then the double dilution method was used for sample solution before be added in to each column from the first row to the eighth row. In the last row, 100 μL of the test solution was thrown into a beaker containing alcohol. The initial compound concentration in each column is 100 μg/mL, the following concentrations were 50, 25, 12.5, 6.25, 3.12, 1.56, 0.78 μg/mL, each compound was set in three parallel experiments and the test values were averaged. The 96-well plates were placed in a 28 °C constant temperature incubator and cultured for 3-4 days. Minimum inhibitory concentration (MIC) was inspected as the lowest concentration in which no fungal growth could be observed. Antibacterial activity was assessed according to the same procedure as antifungal bioassay with the positive drug Chloramphenicol. Cultures were grown for 24 h at 37 ± 0.5 °C in the dark without shaking. Targeted microbes were cultivated in LB medium (yeast extract 5 g/L, peptone 10 g/L, NaCl 10 g/L, pH 7.4) overnight at 37 °C, and diluted bacterial suspension (10^6 CFU per milliliter) for test. All the experiments were performed in three independent replicates.

Anti-Inflammatory Activity

The human monocyte cell line THP-1 (Cell Bank of China Science Academy, Shanghai, China) and *Propionibacterium acnes* (ATCC6919, Xiangfu biotech, Shanghai, China) were used in anti-inflammatory experiments. THP-1 cells were cultured in RPMI1640

medium with 10% fetal bovine serum (FBS, Gibco, NY, USA) in a humidified incubator (37°C, 5% CO_2). *P. acnes* was incubated in an anaerobic environment in a cooked meat medium (Qingdao Ruishui Biotechnology Company, Japan) containing cooked beef pellets. THP-1 cells were stimulated by P. acnes and harvested in the exponential phase. The viability of THP-1 cells was evaluated by MTT analysis. Specifically, THP-1 cells were seeded in a 96-well plate at a density of 2×10^5 cells/well and treated with serially diluted compounds for 36 h (37 °C, 5% CO_2). After that, 20 μL of MTT reagent (5 mg/mL, Genetimes Technology Inc., Shanghai, China) was added to each well and incubated the sample at 37 °C for 4 h. Next, the supernatant was removed and the formazan crystals were completely dissolved in DMSO (150 μL). The absorbance was measured at 570 nm and 630 nm. Tretinoin was used as a positive drug. All the experiments were performed in three independent replicates.

Anti-Inflammatory Activity

CCTGCGGAAGGATCATTACTGAGTGAGGGTCCCTCGGGGCCCAACCTCCCA
CCCGTGTATACCGTACCTTGTTGCTTCGGCGGGCCCGCCGCGCAAGCGGCC
GCCGGGGGGGCGTCAAACCCCCCTCCCTAGGCGAGCGCCCGCCGGAGAC
ACCAACGTGAACACTGTCTGAAGTTTGTTGTCTGAGTTCGATTGTATCGC
AATCAGTTAAAACTTTCAACAATGGATCTCTTGGTTCCGGCATCGATGAAG
AACGCAGCGAAATGCGATAATTAATGTGAATTGCAGAATTCAGTGAATCAT
CGAGTCTTTGAACGCACATTGCACCCCCTGGTATTCCGGGGGGTATGCCTG
TCCGAGCGTCATTGCTGCCCTCAAGCACGGCTTGTGTGTTGGGTCGTCGTC
CCCCCGGGGACGGGCCCGAAAGGCAGCGGCGGCACCGCGTCCGGTCCTC
GAGCGTATGGGGCTTTGTCACCCGCTCTTGTAGGCCCGGCCGGCGCTGGCC
GACGCTGAAAAGCAACCAACTATTTCTCCAGGTTGACCTCGGATCAGGTAG
GGATACCCGCTGAACTTAAGCATAT The 18S rDNA was closest to that of *Aspergillus sp*. LH5 (accession number HQ832844.1) with 100% sequence identity.
